# Isolation and Genome Analysis of an Amoeba-Associated Bacterium *Dyella terrae* Strain Ely Copper Mine From Acid Rock Drainage in Vermont, United States

**DOI:** 10.3389/fmicb.2022.856908

**Published:** 2022-05-23

**Authors:** Lesley-Ann Giddings, Kevin Kunstman, Bouziane Moumen, Laurent Asiama, Stefan Green, Vincent Delafont, Matthew Brockley, Ascel Samba-Louaka

**Affiliations:** ^1^Department of Chemistry, Smith College, Northampton, MA, United States; ^2^Department of Chemistry and Biochemistry, Middlebury College, Middlebury, VT, United States; ^3^Research Resources Center, University of Illinois at Chicago, Chicago, IL, United States; ^4^Laboratoire Ecologie et Biologie des Interactions, Université de Poitiers, UMR7267, Poitiers, France

**Keywords:** amoeba-associated bacterium, amoebae, *Stemonitis* sp., *Dyella terrae*, acid rock drainage

## Abstract

Protozoa play important roles in microbial communities, regulating populations *via* predation and contributing to nutrient cycling. While amoebae have been identified in acid rock drainage (ARD) systems, our understanding of their symbioses in these extreme environments is limited. Here, we report the first isolation of the amoeba *Stemonitis* from an ARD environment as well as the genome sequence and annotation of an associated bacterium, *Dyella terrae* strain Ely Copper Mine, from Ely Brook at the Ely Copper Mine Superfund site in Vershire, Vermont, United States. Fluorescent *in situ* hybridization analysis showed this bacterium colonizing cells of *Stemonitis* sp. in addition to being outside of amoebal cells. This amoeba-resistant bacterium is Gram-negative with a genome size of 5.36 Mbp and GC content of 62.5%. The genome of the *D. terrae* strain Ely Copper Mine encodes *de novo* biosynthetic pathways for amino acids, carbohydrates, nucleic acids, and lipids. Genes involved in nitrate (1) and sulfate (7) reduction, metal (229) and antibiotic resistance (37), and secondary metabolite production (6) were identified. Notably, 26 hydrolases were identified by RAST as well as other biomass degradation genes, suggesting roles in carbon and energy cycling within the microbial community. The genome also contains type IV secretion system genes involved in amoebae resistance, revealing how this bacterium likely survives predation from *Stemonitis* sp. This genome analysis and the association of *D. terrae* strain Ely Copper Mine with *Stemonitis* sp. provide insight into the functional roles of amoebae and bacteria within ARD environments.

## Introduction

Acid rock drainage (ARD) is one of the main sources of surface water pollution worldwide (Dean et al., 2013). This acidic, metal-rich water results from the exposure of sulfide minerals, largely due to mining, to water and oxygen (Nordstrom et al., 2015). The oxidative dissolution of metal sulfides (e.g., pyrite or FeS_2_) forms hydronium and sulfate ions, lowering the pH of water that drains mines and mine waste, thereby increasing the solubility of metal ions (Park et al., 2019). The accumulation of toxic levels of acid and metal ions in this drainage can pollute nearby bodies of water (Kefeni et al., 2017), posing a threat to the environment and human health (Rezaie and Anderson, 2020). This oxidation process is further accelerated by the presence of iron-oxidizing bacteria, which thrive in these acidic environments containing reduced sulfur and iron (Baker and Banfield, 2003; Chen et al., 2016). While ARD microbial communities are dominated by prokaryotes (Méndez-García et al., 2015), single-celled eukaryotes (e.g., protists and fungi) are present and involved in regulating the microbial population as well as the biogeochemical cycling of metals, carbon, nitrogen, and sulfur in these extreme ecosystems (Baker and Banfield, 2003; Aguilera, 2013). Most unicellular eukaryotes that inhabit these environments belong to the following supergroups: *Excavata* (protists), *Archaeplastida* (plants), *Ophisthokonta* (fungi/metazoa), as well as SAR (*Stramenophiles* + *Alveolates* + *Rhizaria*; protists; Chen et al., 2016).

Free-living amoebae (FLA) are ubiquitous in diverse waters, soils, and man-made environments, including ARD (Amaral-Zettler, 2013; Johnson and Aguilera, 2015; Samba-Louaka et al., 2019). These protists are secondary grazers that feed on bacteria and digest them *via* phagocytosis, influencing the composition of the microbial community, mineralization, and nutrient cycles (Dunn et al., 2018; Samba-Louaka et al., 2019). ARD FLA graze on acidophilic bacteria, affecting the oxidation rates of metal sulfides (Johnson and Rang, 1993). These amoebae can also form symbiotic relationships with bacteria, protecting them from harsh environmental conditions as well as providing nutrients or genes that confer a selective advantage, increasing bacterial virulence (Baker et al., 2003; Bertelli and Greub, 2012). Some bacteria have evolved resistance to FLA lysis mechanisms and survive by escaping amoebal phagosomes *via* secretion systems (types I–IV and VII) or by modifying phagosomal vacuoles, subverting antimicrobial mechanisms (Greub and Raoult, 2004; Strassmann and Shu, 2017). However, the roles of amoebae and their symbioses with amoeba-resisting bacteria remain to be fully elucidated within ARD ecosystems.

**Figure 1:**
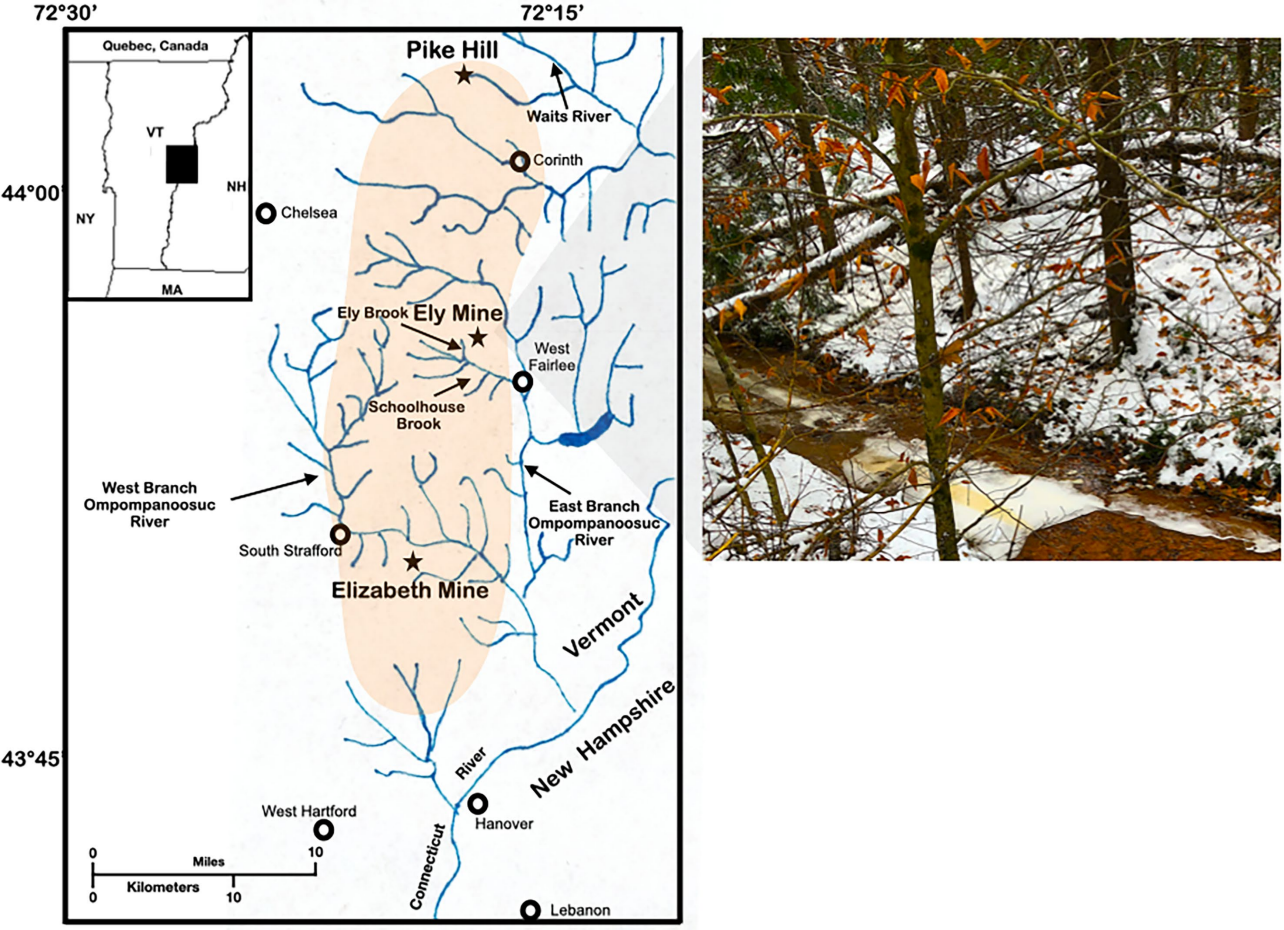
Study site. Map and photograph of Ely Brook (EB-90M) at Ely Copper Mine Superfund site in Vershire, VT on 15 November 2018.

We previously characterized the microbiome and transcriptome of the microbial community in ARD at Ely Brook, which drains the mine tailings at Ely Copper Mine Superfund site in Vershire, VT (Figure 1; Giddings et al., 2020a,b). While acid-tolerant *Proteobacteria* dominated this microbial community, several amoebae were identified in the Ely Brook metagenome (Giddings et al., 2020a). Herein, we aimed to isolate and sequence the whole genome of some of the identified microorganisms. Using cell culture as well as Nanopore and Illumina sequencing, we report the isolation of the slime mold *Stemonitis* sp. from Ely Brook as well as the complete genome of an associated bacterium, *Dyella terrae* strain Ely Copper Mine. This is the first isolation of *Stemonitis* sp. from an ARD environment. Using the annotated *Dyella* genome, potential ecological roles and interactions between the amoeba *Stemonitis* sp. and bacterium *D. terrae* strain Ely Copper Mine in the Ely Brook microbiome are discussed.

## Materials and Methods

### Study Site and Sample Collection

On November 15th, 2018, Ely Brook (43°55′9″ N, 72°17′11″ W), 90 m upstream from the mouth of the brook (abbreviated EB-90M; Figure 1), was sampled by collecting 2 L of water in two autoclaved glass containers, which were stored at 4°C. Three surface water samples were collected in 120-ml high-density polyethylene containers (HDPE Packers, Thermo Fisher; Waltham, MA, United States) for dissolved and total trace-metal analyses. Additional water was collected and either filtered through a 0.22 μM syringe filter (Millex-GS Syringe Filter Unit, MilliporeSigma; Burlington, MA, United States) and/or preserved with sulfuric acid (for organic carbon analyses using standard methods, 5310B and 5310C; American Public Health Association, 2018) in six 40-ml amber borosilicate vials. Three water samples were also collected and preserved with nitric acid for sulfate, nitrite, and nitrate analyses using the Environmental Protection Agency method 300.0 (Pfaff, 1993). The physicochemical properties of samples were either analyzed on-site or in a laboratory.

### Physicochemical Characterization of EB-90M Water

Surface water was analyzed for a variety of field parameters. The pH and temperature of water were monitored using a portable Hannah HI98194 multimeter with select probes. Water was stored at 4°C and analyzed in triplicate for sulfate, nitrate, nitrite, nitrogen contained in organic substances as well as dissolved organic carbon, and total organic carbon within 48 h of collection. Samples were analyzed in Middlebury College’s trace-metal laboratory, except for samples analyzed for sulfate, nitrate, nitrite, total organic carbon, and dissolved organic carbon, which were analyzed at Endyne, Inc. (Williston, VT, United States).

### Inductively Coupled Argon Plasma Mass Spectrometry Analysis of Water

A Thermo Fisher inductively coupled argon plasma mass spectrometry (ICAP-MS) was used to test three water samples for dissolved and total trace-metal concentrations using internal and external standards. The instrument was tuned using the THERMO-5A (Inorganic Ventures; Christiansburg, VA, United States) multielement standard according to the manufacturer’s instructions. The 2008ISS multielement standard (Inorganic Ventures; Christiansburg, VA, United States) was used as an internal reference for all samples at a concentration of 100 ppb to correct for instrument drift. All water samples, including the standards, were spiked with the internal reference. See the Supplementary Material for the instrument parameters and standards used.

### Isolation of Amoebae From Ely Brook Water

Water (100 ml) was filtered through 3-μm nitrocellulose filter paper by vacuum filtration. The resulting filter paper was placed upside down on non-nutrient agar (sodium chloride, 60 g/L; sodium citrate tribasic, 0.8 g/L; magnesium sulfate, 4 mM; monobasic sodium phosphate, 2.5 mM; monobasic potassium phosphate, 2.5 mM; calcium chloride, 0.5 mM; and agar, 15 g/L) plates seeded with 10^4^ *Escherichia coli* K12 strain SMG 123 (PTA-7555, ATCC; Manassas, VA, United States) cells and incubated at 20°C. Plates were examined under an inverted microscope every 3 days to probe for amoebae along the edges of filter paper. Once amoebae were detected, they were recovered by scraping and transferred to a Page’s Amoeba Saline Solution (PAS) (magnesium sulfate, 4 mM; calcium chloride, 0.4 M; sodium citrate dihydrate, 0.1%; monosodium phosphonate, 2.5 mM; monopotassium phosphonate, 2.5 mM; and pH adjusted to 4) supplemented with 5 × 10^8^ *E. coli* cells in Luria-Bertani medium for growth at 20°C. Amoebae were subcultured by dilution in PAS once they reached confluency. Regarding light microscopic observations, cultures of amoebae were transferred into 35 mm dishes with glass coverslips (μ-dish, Ibidi; Gräfelfing, Germany). Amoebae were left to adhere at room temperature and observed using an inverted microscope equipped with differential interference contrast (DIC) (Olympus; Tokyo, Japan). The contrast and brightness of images were adjusted further using ImageJ (Abramoff et al., 2004).

### DNA Extraction, 18S rRNA Sequencing, Library Construction, and Whole-Genome Sequencing

The amoebal liquid culture (30 ml) was filtered through a 3-μm nitrocellulose filter paper and DNA was extracted using the High Pure PCR template preparation kit (Roche; Basel, Switzerland) followed by ethanol precipitation. Phusion DNA polymerase (Thermo Fisher; Waltham, MA, United States) along with 200 μM deoxynucleotide triphosphates, 0.5 μM 566F (5ʹ-CAGCAGCCGCGGTAATTCC-3ʹ) and 1200R (5ʹ-CCCGTGTTGAGTCAAATTAAGC-3ʹ) primers (Hadziavdic et al., 2014) were used to amplify the 18S rRNA gene from 183 ng of extracted DNA in a total volume of 25 μl using the following thermocycling parameters: 98°C for 30 s, followed by 35 cycles of 98°C for 10 s, 50°C for 20 s, 72°C for 1 min, and a final extension at 72°C for 7 min. Amplicons (~750 bp) were sequenced by the Sanger method, and the taxonomy was determined by comparing the sequence to those in the NCBI nr database using BLAST (Altschul et al., 1990).^1^ The partial 18S rRNA sequence was deposited to GenBank under the accession number OL589616.1.

For whole-genome sequencing of the isolated amoeba, library preparation and sequencing were performed at the University of Illinois at Chicago Sequencing Core. Nanopore sequencing libraries were generated from genomic DNA using the Oxford Nanopore genomic DNA library protocol SQK-LSK109 according to the manufacturer’s instructions (Oxford Nanopore Technologies). Sequencing was performed using a FLO-MIN106 (R10 Version) flow cell on a GridION sequencer. An Illumina library was prepared using the Nextera FLEX Workflow (#20018705 Illumina Inc. San Diego, CA, United States) with 100 ng template and 5 cycles of PCR according to the manufacturer’s instructions. The library was quantified using a Qubit DNA High Sensitivity kit (Life Technologies, #Q32851, Grand Island, NY, United States), and size distribution was assessed using an Agilent 4200 TapeStation System (Agilent Technologies, G2991AA, Santa Clara, CA, United States) using TapeStation D5000 ScreenTape, ladder, and assay (Agilent Technologies, # 5067–5588, 5067–5590 and 5067–5589, Santa Clara, CA, United States). The library was finally pooled and run on an Illumina MiSeq instrument using MiSeq Reagent Nano Kit, v2 (300 cycles) (Illumina, TG-142-1,001, Foster City, CA, United States) for quality control and library balancing purposes. A new pool was made based on the MiSeq run results, quantified as described above, and sequenced on an Illumina NextSeq 500 Instrument in paired end mode (2 × 150 bp) (Illumina, FC-404-2004, Foster City, CA, United States) with a 1% phiX spike-in.

### Data Preprocessing and Genome Assembly

Raw data from Nanopore sequencing were basecalled using Guppy 3.6.1 (Oxford Nanopore Technologies; Oxford, United Kingdom) and data quality control was performed with pycoQC v2.5.0.14 (Leger and Leonardi, 2019). Porechop V 0.2.4 (Wick and Holt, 2021) was then used to filter and remove adapters from the basecalled data. Flye 2.8 (Kolmogorov et al., 2019, 2020) was used to make the assembly, which was benchmarked using both single-genome and meta modes and the following parameters: -min-overlap 1, 1.5 kb; and the default as well as -iterations 2, 5, and 10. Another assembler, Canu (Koren et al., 2017), was used to validate the assembly from Flye using the following parameters: minReadLength = 1,000 -minOverlapLength = 500 -nanopore-raw.

### Genome Polishing and Busco Analyses

The genome was self-polished by Medaka v 0.8.1 (Oxford Nanopore Technologies, 2018) with the Nanopore reads used for the assembly. Once the assembly was self-polished, Illumina data was used to polish the genome. Illumina data underwent the standard preprocessing steps and FastQC v0.11.9 (Andrews, 2010) was used to check the read quality. Adapters and reads of poor quality were filtered by Fastp v. 0.21.0 (Chen et al., 2018). Contaminating reads were filtered using a mapping approach targeting the human and *E. coli* genomes using Bowtie2 (Langmead and Salzberg, 2012). The self-polished genome was polished using Polypolish v0.4.3 (Wick and Holt, 2021). To assess the quality of the assembly, Busco v 5.0.0 (Manni et al., 2021) was used with the following three levels of taxonomy: phylum (proteobacteria_odb10), class (gammaproteobacteria_odb10), and order (xanthomonadales) with 219, 366, and 1,152 ortholog groups, respectively.

### Bacterial Identification and Genome Annotation

Blastn (Altschul et al., 1990) was used to compare the largest contig against the NCBI nt database for phylogenetic classification. The raw genome was annotated by Prokka v 1.14.6 (Seemann, 2014) using default parameters. Once the bacterial taxonomy was confirmed, a protein database containing 169,481 *Dyella* sp. proteins from NCBI was constructed and used to annotate the coding sequences from the assembled genome. Ribosomal RNA gene sequences were extracted from the annotation and used to accurately identify the genus and species using the EzBioCloud platform (Yoon et al., 2017).

### Phylogenetic Analysis

To determine the phylogenetic position of the associated bacterium, a representative set of 16S rRNA gene sequences were compiled from the non-redundant nucleotide database of the NCBI. A total of 24 full or near-full length sequences, including the complete 16S rRNA sequence for the bacterium, were collected, manually inspected, and aligned using MUSCLE v3.8.31 (Edgar, 2004), resulting in a global alignment of 1,542 nucleotide positions. ModelFinder (Kalyaanamoorthy et al., 2017) was used to determine the best substitution model for phylogenetic inferences. Based on the best Bayesian information criterion score, the HKY + F + I model was selected. Inference of the phylogeny was done by maximum likelihood using IQ-TREE v 2.0.3 (Minh et al., 2020). The robustness of the inference was tested by further applying 1,000 iterations of conventional bootstraps and SH-approximate likelihood ratio tests. The resulting tree was rendered in FigTree v 1.4.4 (Rambaut, 2021).

### Fluorescence *in situ* Hybridization Experiments With Confocal Microscopy

Isolated amoebae (10^5^ cells per well) were seeded on a microscope slide 10-well (Thermo Scientific Cel-Line® Brand; Waltham, MA, United States). Cells were fixed using PAS-4% paraformaldehyde for 15 min at room temperature. After the removal of the fixing solution, the slide was stored at −20°C.

FISH probes were designed based on 16S and 18S rRNA sequences recovered from the isolates from this study. Candidate probes were further tested *in silico* using Decipher (Wright, 2013) and Mathfish (Yilmaz et al., 2011) to validate their specificity and theoretical formamide curves. Ultimately, specific probes, Stemonitis_383 (5ʹ-Cy5-TTCACCACTAGCCCGGC-3ʹ) and Dyellaterrae_176 (5ʹ-Cy3-CCAACCGCGCAAGGCCC-Cy3-3ʹ), were used in addition to the eubacterial probes, EUB338 I-III (5ʹ-FITC-GCTGCCTCCCGTAGGAGT-FITC-3ʹ; 5ʹ-FITC-GCACCCACCCGTAGGTGT-FITC-3ʹ; and 5ʹ-FITC-GCTCCCACCCGTAGGTGT-FITC-3ʹ) at concentrations of 0.5 mM. *In situ* hybridization was performed according to a previously published protocol, freely available at the website of the SILVA database^2^ (standard FISH protocol). Optimal formamide concentration for achieving FISH was experimentally estimated to be 20%. After hybridization, all samples were mounted using Citifluor™ AF1 plus 4′,6-diamidino-2-phenylindole (DAPI). Samples were examined with a laser scanning confocal microscope (Olympus FV3000; Tokyo, Japan). Laser lines (405, 488, 561, and 640 nm) were used for the excitation of DAPI, fluorescein isothiocyanate (FITC), cyanine 3, and cyanine 5, respectively. Emission fluorescence was recorded through spectral detection channels between 430 and 470 nm (blue), 500 and 540 nm (green), 570 and 620 nm (red), and 650 and 750 nm (far red) fluorescence emission. Images (1,024 × 1,024 pixels) were acquired with UPLAPO100XOHR NA:1.50 objective lens and x2 numerical zooming (0.06 μm pixel size). A 3-dimensional optical sectioning of 0.2 was driven with a step Z-axis motor. Images were analyzed for 3-dimensional rendering with Imaris software (Bitplane; Zürich, Switzerland).

### Functional Annotations and Analysis of Bacterial Genes Involved in Lignin Degradation, Natural Product Biosynthesis, and Metal Resistance

Open reading frames (ORFs) of the associated bacterium were annotated by the Kyoto Encyclopedia of Genes and Genomes (KEGG) automatic annotation server (KAAS)^3^ using prokaryote and bidirectional best hit parameters as the assignment method (Kanehisa and Goto, 2000). KAAS functionally annotated genes using BLAST comparisons to a curated KEGG database were used to identify KEGG orthologs (KOs) and metabolic pathways. KOs (or K numbers) were then assigned to KEGG pathways and biomolecular reaction pathways for information transfer and expression (BRITE) hierarchies. ORFs were also annotated *via* the RAST web annotation tool version 2.0 (Aziz et al., 2008) using the RasTtk annotation pipeline to identify lignocellulose decomposition-related enzymes for comparison with other publicly available *Dyella* genomes, including *D. terrae* KACC 12748 (BioProject: PRJNA523522), *D. jiangningensis SBZ 3-12* (BioProject: PRJEB16000), and *D. jiangningensis* FCAV SCS01 (BioProject PRJNA386033), a species speculated to be involved in lignin degradation (Desiderato et al., 2018; Constancio et al., 2020). While only the *D. jiangningensis SBZ 3-12* genome was complete, the other genomes had a completeness of 100%, according to the checkM metrics database (Parks et al., 2015).

Prokka-annotated ORFs involved in lignin degradation were compared against the Laccase Engineering Database (LccED; Sirim et al., 2011), which contains curated entries for genes involved in lignin metabolism. HMMER v. 3.1b (Wheeler and Eddy, 2013) profiles from families of LccED were generated and used to annotate predicted ORFs. If a sequence was annotated in multiple LccED families with an e-value less than 1E-5, then the annotation that gave the lowest e-value was kept. False positives were detected using CD-Search (Marchler-Bauer and Bryant, 2004) to ensure all genes had copper-binding domains. All matches had e-values below 1E-5. The genome was then mined for secondary metabolite biosynthetic gene clusters (BGCs) using the bacterial AntiSMASH 6.0 (Blin et al., 2021) web server. Default parameters and the following features were used to identify BGCs: knowncluster blast, subcluster blast, and active site finder. DIAMOND (Buchfink et al., 2015) blastp was used to mine the genome for metal resistance genes by querying genes against 285 experimentally validated metal resistance genes (a total of 419 sequences) in the BacMet database (Pal et al., 2013). Using the “more sensitive” mode, DIAMOND (Buchfink et al., 2015) annotated translated protein sequences with a maximum target sequence of 15 and e-values below 1E-5. The genome was also mined for antibiotic resistance genes that were within proximity or colocalized with BGCs using Antibiotic Resistant Target Seeker (ARTS) version 2 (Mungan et al., 2020) using default parameters. Duplication and BCG proximity, resistance model screens, and genomes that mapped to the following phylum was selected: Gammaproteobacteria.

### Data Sharing and Nucleotide Accession Numbers

Raw sequence data, assembled genome, and metadata were submitted to the National Center for Biotechnology Information and are accessible under BioProject PRJNA771916.

## Results

### Physicochemical Properties

The temperature and pH of sampled water at EB-90 M were 0.7°C and 3.93, respectively. Water sulfate levels (127 ± 6 mg/L) were within EPA-recommended concentrations (<250 mg/L; United States Environmental Protection Agency, 2003). Most nutrients, including nitrate and nitrite (<0.02 mg/L) were below the detection limit. Low levels of total (1.8 ± 0.3 mg/L) and dissolved organic carbon (1.5 ± 0.3 mg/L) were detected in water in addition to high metal concentrations. The most abundant elements in water were Mg, Al, and Fe (3.00–4.05 mg/L; Table 1), and the amounts of total and dissolved elements were similar across water samples.

**Table 1:** Chemical composition of EB-90M water.

Element	Dissolved	Total
Na	1.24	1.25
Mg	3.08	3.00
Al	3.00	3.06
Cr	<0.01	<0.01
Mn	0.255	0.279
Fe	3.44	4.05
Co	0.0606	0.0669
Ni	0.0233	0.0264
Cu	1.74	1.84
Zn	2.82	3.11
As	0.384	0.289
Cd	<0.01	<0.01
Sb	<0.01	<0.01
Ba	<0.01	0.0104
Pb	<0.01	<0.01

Selected elemental analysis for dissolved and total elements in EB-90M water in mg/L. All values were determined in triplicate by ICAP-MS.

### Isolation of Amoeba and Associated Bacterium at EB-90M

**Figure 2:**
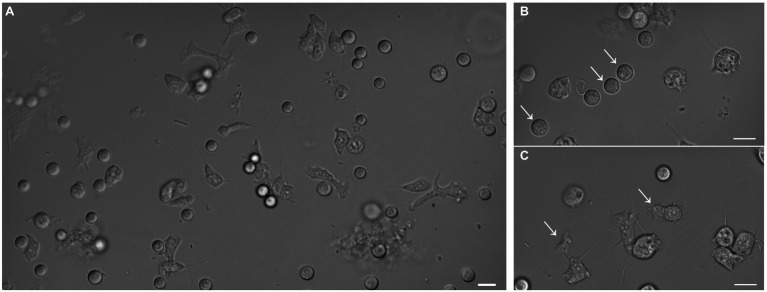
Differential interference contrast micrographs of isolated amoeba. Images show morphology features highlighting the diversity of shapes and sizes of the isolated amoeba. **(A)** Global view of the different shapes of amoebae. Panels **(B)** and **(C)** focus on rounded (arrows) and flagellated (arrows) amoebae, respectively. The bar length represents 10 µm for all panels.

Amoebae that grew in *E. coli*-PAS were recovered from the filter grown on *E. coli*-non-nutrient agar plates. After several subcultures, microscopic observations revealed the isolation of at least two different forms of the same amoeba, rounded and flagellated (Figure 2). The latter had one or two flagella from one pole of the cell, which were roughly 7 μm in diameter. Similar to *Stemonitis fusca*, we observed transparent, rounded, and irregular shaped flagellated protoplasts 7.5–10 μm in length (Dai et al., 2020). The 18S rRNA gene sequence had a best hit (e-value = 2E-120) corresponding to a member of the Amoebozoa supergroup, *Stemonitis* sp., with 99.79% identity (see phylogeny in Supplementary Figure 1). These genetic and microscopic data provide strong evidence that the isolated amoeba was *Stemonitis* sp.

**Figure 3:**
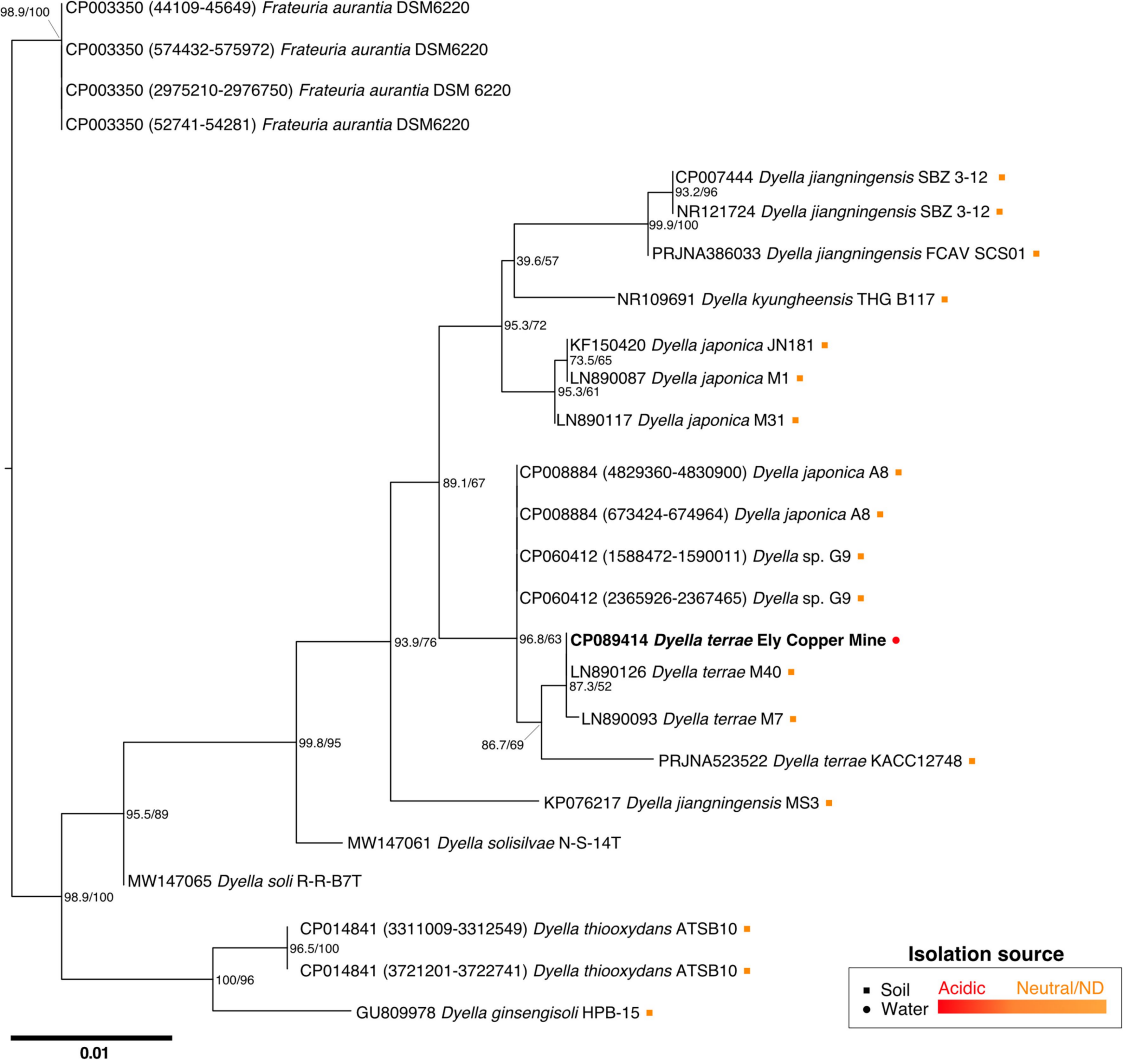
Maximum likelihood tree based on near-complete 16S rRNA gene sequences showing the phylogeny of *Dyella* sp. Sequences of *Frateuria* were used as an outgroup. Bootstrap **(left)** and SH-like approximate likelihood ratio **(right)** support values are expressed in percentages at nodes. Bar represents 0.01 substitutions per nucleotide position. Circle and square symbols represent species isolated from water and soil, respectively. The colors of the symbols indicate whether the environment was acidic (red) or either neutral/no pH was reported (orange). Isolates without associated metadata have no symbol.

While attempts to sequence and characterize the *Stemonitis* genome are incomplete, we did obtain a complete bacterial genome instead, for which the best hit for the 16S rRNA gene sequence corresponded to the bacterium *D. terrae*. The full-length 16S rRNA sequence of the *Dyella* isolate is closely related to *D. terrae* M40 and M7, with high sequence similarities of 99.02% and 99.93%, respectively (Figure 3). We refer to this bacterium as *D. terrae* strain Ely Copper Mine, which appears to be associated with *Stemonitis* sp. after successive generations in PAS. Phylogenetic inference conducted by maximum likelihood confirmed the close phylogenetic relationship of the isolate with representative *D. terrae* species from various locales (Figure 3). Based on its phylogeny and the presence of liposaccharide biosynthesis, assembly, and transport proteins, we inferred that *D. terrae* strain Ely Copper Mine is Gram-negative (Bertani and Ruiz, 2018).

### FISH Experiments

**Figure 4:**
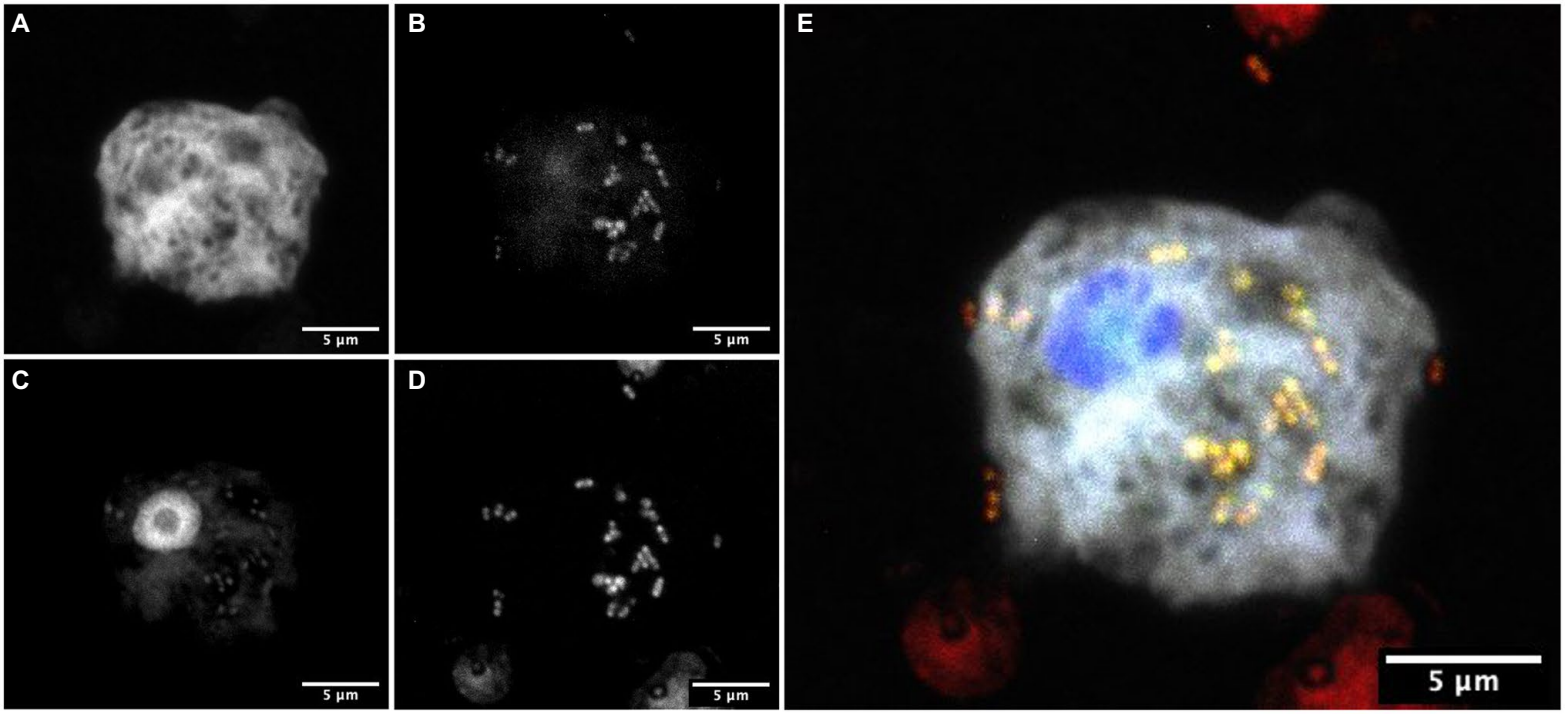
FISH showing *Dyella* in association with *Stemonitis* sp. FISH was performed using **(A)** a *Stemonitis*-specific Cy5 probe; **(B)** EUB3338 I-III FITC probes for labeling eubacteria; **(C)** DAPI; and **(D)** a *Dyella*-specific probe Cy3 probe. **(E)** The composite image of all labeling highlights the superimposition of eubacteria- and *Dyella*-specific FISH. The bar length represents 5 μm for all panels.

**Figure 5:**
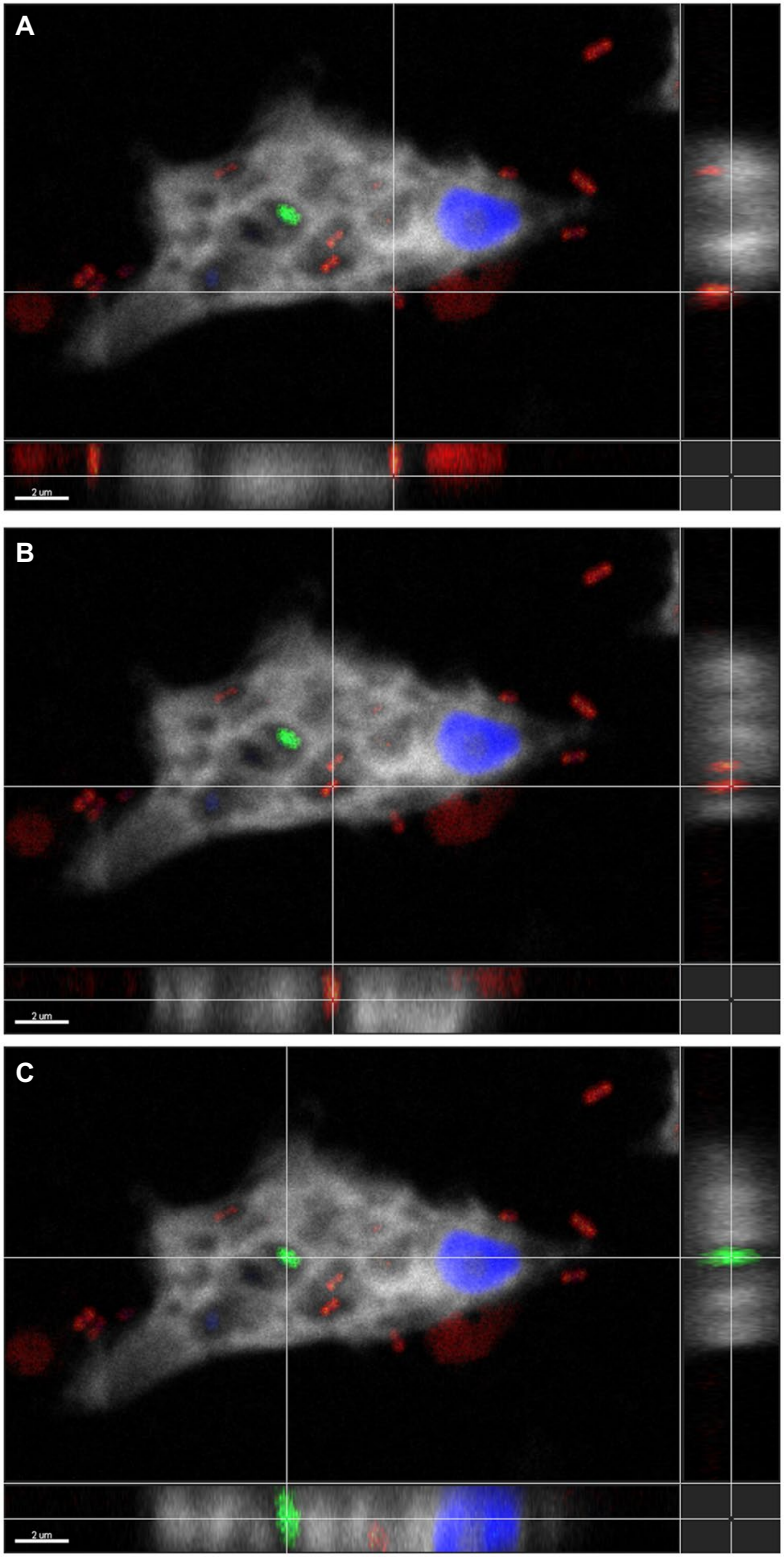
Three-dimensional FISH analysis of *D*. *terrae* strain Ely Copper Mine inside Stemonitis sp. FISH microscopy images were analyzed in-depth to determine the extracellular (panel **A**) and the intracellular (panel **B**) location of *Dyella* (in red) and other bacteria (in green, panel **C**). The margin images underneath and to the right of panels **(A-C)** represent projections through x-y and z planes, respectively. The colors white and blue correspond to the cy5 probe against *Stemonitis* and DAPI, respectively. The bar length represents 2 µm for all panels.

To confirm the association between *D. terrae* strain Ely Copper Mine and *Stemonitis* sp., we performed a FISH experiment with bacterial 16S rRNA probes. We observed the presence of bacteria inside and nearby cells of *Stemonitis* sp. (Figures 4, 5). The in-depth, 3-dimensional analysis showed *D. terrae* strain Ely Copper Mine inside *Stemonitis* vacuoles (Figure 5). We also found other bacteria stained by the eubacteria probe (not the *Dyella* probe) inside *Stemonitis* (Figures 4, 5). Given that the *Dyella* was identified by two probes, a *Dyella*-specific probe and eubacterial probe, Figures 4, 5 also demonstrate that other non-*Dyella* eubacteria were associated with this strain of *Stemonitis*. These non-*Dyella* bacteria could be other environmental bacteria or the *E. coli* food source for *Stemonitis* sp.

### Amoeba-Associated Bacterial Genome

Sequencing resulted in 949,080 raw Nanopore reads, which were filtered to 800,193. The reads were assembled using Flye in single-genome mode, resulting in six contigs. Two contigs had no associated phylogeny and three were shown to contain sequences corresponding to amoebal RNA and mitochondrial DNA according to NCBI nt, NR, and RNAmmer. The remaining contig, the focus of this study, had 5.36 Mbp of circular DNA with 543x coverage and 62.5% GC content. Protein coding genes (4,841) were identified as well as six rRNA-, 49 tRNA-, one tmRNA-, and 25 miscellaneous RNA-coding genes. When genes were compared against the NCBI nt database, most hits belonged to the *Dyella* genus. KEGG assigned 2,149 (44%) out of 4,841 protein coding genes to K numbers (KO identifiers), some of which were indicative of assimilatory sulfate reduction (e.g., *cysHIJ*; see Supplementary Table 3), nitrate reduction (*napAB*; K02568), and the oxidation of aromatic carbons found in lignin (*gst*; K00799). Genes were assigned to KEGG pathways (273), including nitrogen and sulfur metabolism and xenobiotic degradation. Annotated genes were then assigned to BRITE hierarchies, a classification system for connections that can exist between genes and proteins in biological systems. Most BRITE level 1-annotated genes were involved in metabolism (505), particularly carbohydrate and amino acid metabolism (Supplementary Tables 4 and 5). Genes were also involved in BRITE hierarchies (395) mainly related to protein families: signaling and cellular processes as well as protein families: genetic information processing (Supplementary Tables 4 and 5). Seventeen genes involved in type IV secretion systems (BR:ko02044; Supplementary Table 6), commonly associated with bacterial resistance to amoebae, were also identified, as well as genes involved in virulence in eukaryotes, including a gene encoding an ankyrin repeat (ANK) domain-containing protein (K10380; BR:ko04812). With the exception of nitrate reduction, similar BRITE level 1-annotated genes and KEGG pathways were found in the previously sequenced *D. terrae* KACC 12748 (Supplementary Tables 4 and 7).

### Genes Involved in Lignin Degradation, Secondary Metabolism, and Metal Resistance

Several genes are putatively involved in lignin degradation, including 26 RAST-annotated hydrolases (Supplementary Table 8), a dye-decolorizing (DyP) peroxidase, and four laccases containing copper-binding domains (HMMER annotation using LccED; Supplementary Table 9; Janusz et al., 2017). Hydrolases, a DyP-type peroxidase, and laccases were also found in the closely related *D. terrae* KACC 12748 genome and strains of *D. jiangningensis* (Supplementary Tables 8–10) characterized as having lignin-degrading capabilities (Desiderato et al., 2018; Constancio et al., 2020). In addition to having lignin-degrading genes, the *D. terrae* strain Ely Copper Mine genome also contained a total of six putative biosynthetic gene clusters (BGCs) dedicated to secondary metabolism (antiSMASH annotation; Supplementary Table 11). The BGCs were involved in the biosynthesis of a polyketide/non-ribosomal peptide, arylpolyene, terpenes, and post-translationally modified peptide products (RiPP) secondary metabolites. All of these BGCs are predicted to produce unknown secondary metabolites. Five BGCs were identified in the genome of *D. terrae* KACC 12748 and not all are predicted to produce the same classes of secondary metabolites (Supplementary Table 11).

Putative metal resistance genes (229) were also identified in the genome, most of which were *copR* (25), *czcR* (12), and *wtpC* (11), which are involved in copper (Mills et al., 1994), zinc/cadmium/cobalt (Perron et al., 2004), and tungsten/molybdenum resistance (Bevers et al., 2006), respectively. Of the 285 experimentally validated metal resistance genes in the BacMet database, 89 unique genes were identified in the genome (Supplementary Table 12). The same analysis of the genomes of *D. terrae* KACC 12748 and other strains of *D. jiangningensis* yielded a similar distribution of metal resistance genes (Supplementary Table 12). Lastly, 37 antibiotic resistance genes were identified, most of which encoded resistance nodulation cell division (RND) efflux pumps (Supplementary Table 13). Antibiotic resistance genes (OTCace, an ornithine/aspartate binding domain; AAC3, an aminoglycoside acetyltransferase) also colocalized with two BGCs involved in the synthesis of terpenes and RiPPs.

## Discussion

Amoebae are reservoirs for bacteria (Shi et al., 2021) and thought to provide nutrients and protect bacteria growing under harsh conditions, but our understanding of their roles and symbioses in ARD remains limited (Baker and Banfield, 2003; Greub and Raoult, 2004; Hilbi et al., 2011). In previously collected metagenomic data sets from ARD in Ely Brook (Giddings et al., 2020a,b), we identified protozoa in EB-90M water and sediment metagenomes. Here, we report the isolation of an amoeba corresponding to the genus *Stemonitis* from acidic, metal-rich EB-90M water. After several passages in PAS, we also identified an associated bacterium belonging to the genus *Dyella* and characterized its genome to understand its role in ARD as well as its unprecedented association with *Stemonitis* sp.

*Stemonitis* spp. are slime molds with unique characteristics, such as a spore-to-spore life cycle, and commonly found on decaying plant matter in terrestrial ecosystems (Stephenson and Stempen, 1994). Depending on growth conditions, they can be found as cellular uninucleate amoeboflagellates, acellular multinucleate plasmodia, and stationary spore-bearing sporocarps (Dai et al., 2020). While we only observed unicellular and flagellated states, the composition of our media could explain the absence of plasmodia and sporocarps, as the formation of plasmodia and sporulation commonly occur on water- or oat-agar surfaces (Dai et al., 2020). Temperature, pH, numbers of cells, and the duration of photoperiods are also important variables that affect how plasmodial slime molds complete their life cycles (Gao et al., 2017; Zhu et al., 2019; Dai et al., 2020). Determining the *Stemonitis* life cycle will be important for further characterization, as species in Myxogastria are classically identified by the structural characteristics of their sporocarps and plasmodia (Clark and Haskins, 2014). These forms are also commonly reported to associate with bacteria, enabling them to fix nitrogen, degrade wood, and produce extracellular enzymes (Kalyanasundaram, 2004).

Interestingly, *D. terrae* strain Ely Copper Mine was observed to have a stable association with unicellular states of the isolated amoeba after successive culturing. *Dyella terrae* belong to the order and family, Xanthomonadales and *Rhodanobacteraceae* (Naushad et al., 2015), respectively, which are involved in sulfur and carbon biogeochemical cycling (Pujalte et al., 2014) and have been found in ARD environments (Gruzdev et al., 2020; Valkanas et al., 2021). In particular, the *Dyella* genus has been detected in the metagenomes of acidic, metal-rich environments (Valentín-Vargas et al., 2018), including EB-90M (Giddings et al., 2020b), with similar physicochemical properties to those of samples collected in this study (Table 1). *Dyella terrae* appears to be acid-tolerant, as this species is reported to thrive at neutral pH (Weon et al., 2009) and most sequenced strains are not from acidic, metal-rich ecosystems (Figure 3). The size of the bacterial genome is similar to those of *Dyella* strains with genomes ranging from 4.0 to 5.49 Mbp (He et al., 2017; Ou et al., 2019). *Dyella terrae* strain Ely Copper Mine does not have a reduced genome, has similar GC content (59.1%–65.2%) to that of other *Dyella* strains (Desiderato et al., 2018; Ou et al., 2019), and can exist outside amoebal cells (Figure 5), indicating that it is likely a facultative symbiont of *Stemonitis* sp. (McCutcheon and Moran, 2012). The bacterium could be in an early stage of association without the necessary evolutionary time or selective pressure for genome reduction (Fisher et al., 2017). Confocal microscopy (Figures 4, 5) confirmed the association between the strains of *Dyella* and *Stemonitis*. This association may be related to “farming behavior” (Kutschera and Hoppe, 2019), in which amoebae, such as *Dictyostelium discoideum*, do not consume all bacteria, but rather store some as a food source by incorporating them into fruiting bodies and then seed them upon arriving to a new location (Brock et al., 2011).

KEGG annotations provided insight into potential roles of *D. terrae* strain Ely Copper Mine in EB-90M water. Assigned KOs indicated the sulfate-reducing ability of *D. terrae* strain Ely Copper Mine, which could be used to biomine or bioremediate ARD sites given the appropriate pH for catalytic activity (Sánchez-Andrea et al., 2014; Chen et al., 2016). Similar genes were identified in the *D. terrae* KACC 12748 genome (Supplementary Table 3). While other species of *Dyella* (*D. japonica*, *D. soli*, and *D. koreensis*) have been reported to reduce nitrate, *D. terrae* sp. nov JS14-6^T^ did not reduce nitrate (Xie and Yokota, 2005; Weon et al., 2009). We also could not identify nitrate reduction genes in the *D. terrae* KACC 12748 genome. However, our analysis of the genome of the *D. terrae* strain Ely Copper Mine showed it has the genetic potential to reduce nitrate as it contains the *napA* gene (K02567; Weon et al., 2009). *Dyella terrae* sp. nov JS14-6 was also previously reported to not hydrolyze carbohydrates, such as α-glucosidase (Weon et al., 2009). Yet, genes involved in the hydrolysis of carbohydrates (K01187; α-glucosidase) and the oxidation of aromatic carbons (K00799; glutathione-*S*-transferase) found in lignin were identified in *D. terrae* strain Ely Copper Mine and *D. terrae* KACC 12748. Thus, given the appropriate environmental conditions, these genes could provide a carbon source, which could be useful in carbon-limited environments, such as EB-90M.

Lignin is a complex polycyclic aromatic hydrocarbon that is a major component of plant biomass. Its hydrolysis, oxidation, and depolymerization plays a critical role in carbon recycling (Kamimura et al., 2017). Several genes encoding peroxidases, hydrolases, and multicopper oxidases (e.g., laccases) involved in this process have been identified in *D. terrae* strain Ely Copper Mine in comparable numbers to those in other lignin-degrading *Dyella* (Supplementary Tables 8–10; De Gonzalo et al., 2016; Desiderato et al., 2018). While the lignin-degrading capabilities of *D. jiangningensis* FCSAV SCS01 were based on several of its genes encoding hydrolases (Desiderato et al., 2018), a *D. jiangningensis* isolate was observed to exhibit extracellular cellulase and amylase enzyme activities involved in lignin degradation (Constancio et al., 2020). Thus, these genes are likely involved in lignin degradation and could influence amoebae growth in carbon-limited EB-90M water, as low concentrations of lignin can be an indicator of high amoebal density (Krashevska et al., 2017, 2018).

Although most closely related strains of *Dyella* have not been isolated from metal contaminated sites (Figure 3), *Dyella* have been detected at other metal-rich sites (Zhao et al., 2013; McGee et al., 2017; Banach et al., 2020). In fact, *D. jiangningensis* XL23 has been used in the remediation of these locales for its metal resistance genes (He et al., 2017). Thus, it is not surprising that *D. terrae* strain Ely Copper mine has several metal resistance genes (30% of 285 experimentally verified BacMet metal resistance genes) involved in resistance to the following metals: copper (*copR*); zinc, cadmium, and cobalt (*czcR*); and tungsten/molybdenum (*wtpC*). These genes have also been detected in *D. terrae* KACC 12748 and *D. jiangningensis* genomes (Supplementary Table 12; Desiderato et al., 2018), and copper resistance genes, including *copABCD*, have also been found in *Dyella* at metal contaminated sites (McGee et al., 2017). These genes are likely important for these metal-tolerant bacteria to survive within this metal-rich environment (Table 1).

In addition to metal resistance and lignin degradation, *D. terrae* strain Ely Copper Mine has the potential to produce new secondary metabolites, as its BGCs are not similar to those involved in the *D. terrae* KACC 12748 genome as well as those involved in making known compounds (e.g., >30% sequence similarity). Two of the BGCs involved in producing RiPPs and terpenes colocalized with antibiotic resistance genes, suggesting that the secondary metabolites produced may have antibacterial activity (Alanjary et al., 2017). Aside from potentially having novel bioactivity, these secondary metabolites may also be involved in the bacterial association with *Stemonitis*. For example, strains of *Dyella* have been shown to produce volatile compounds (i.e., terpenes) that impact amoebal growth (Schulz-Bohm et al., 2017). Terpenes play a role in mediating bacterial-protist interactions and there are two BGCs in the *Dyella* genome dedicated to making terpenes (Supplementary Table 11).

Several amoebae resistance genes, such as those involved in secretion systems, were also found in the *D. terrae* strain Ely Copper Mine genome. The type IV secretion system is one of the most common mechanisms of resistance against amoebae (Christie, 2004). This secretion system transfers proteins and DNA, facilitating horizontal gene transfers. The *D. terrae* strain Ely Copper Mine genome contains a virB/D4 type IV secretion system, including *virB2*, *virB3*, *virB4*, virB9, *virB10*, *virB11*, *virD2*, and *virD4* genes, some of which are clustered together. These are secretion P-type DNA transfer genes that aid in substrate secretion through a cell envelope spanning structure, preventing bacterial cell lysis and encouraging cell growth (Christie, 2004). *Dyella terrae* strain Ely Copper Mine may have evolved these mechanisms as an adaptive response to predation pressure, enhancing its persistence (Hoque et al., 2021). Other virulence genes were also found in the genome, including those encoding an ANK-containing protein, which has unclear origins but may be acquired from eukaryotes *via* horizontal gene transfer or result from convergent evolution (Al-Khodor et al., 2010). ANK-containing proteins are involved in bacterial virulence and commonly found in eukaryotes, mediating protein–protein interactions within host-cell metabolism (Moliner et al., 2010). Similar virulence genes, such as *virB* and those encoding an ANK-containing protein, were also identified in the *D. terrae* KACC 12748 genome. These genes may be key to *D. terrae* strain Ely Copper Mine occupying *Stemonitis* sp.

While the association between *Dyella* and *Stemonitis* has not been reported before, *Dyella* have been shown to affect the performance of protozoa, such as *Vermamoeba* and *Saccamoeba*, by reducing their trophic activity, motility, and growth (Schulz-Bohm et al., 2017). Protozoa-associated prokaryotes commonly serve as electron sinks (e.g., sulfate reduction; Ohkuma et al., 2015), provide chemical defense against predators (e.g., secondary metabolites; Görtz, 2006), and/or provide nutrition for the host cell (Gast et al., 2009). *Dyella terrae* strain Ely Copper Mine could function in all three roles, especially being a nutrient source for the host cell in this nutrient-limited environment. Alternatively, the isolated *Stemonitis* could simply serve as a suitable growth environment (e.g., being neutrophilic) for acid-tolerant bacteria conferring resistance to predation (Baker et al., 2003). The presence of other bacteria within *Stemonitis* (Figures 4, 5) could also influence these interactions. While additional experiments are required to further explore the association between these strains of *Dyella* and *Stemonitis*, *D. terrae* strain Ely Copper Mine likely plays many roles within the ARD community, including biomass production *via* lignin degradation, metal resistance, and the biosynthesis of secondary metabolites.

## Data Availability Statement

The datasets presented in this study can be found in the supplementary material and the National Center for Biotechnology Information BioProject database, www.ncbi.nlm.nih.gov/bioproject (project ID PRNAJ771916). The partial 18S rRNA gene sequence has been deposited within GenBank under the accession number OL589616.1. Further inquiries can be directed to the corresponding author.

## Author Contributions

L-AG, AS-L, and SG were involved with conceptualizing the methodology and designing the study. AS-L, L-AG, KK, LA, and VD performed the field work and the experiments. AS-L, L-AG, BM, and MB analyzed, interpreted, and curated the data. L-AG procured funding for the project and wrote the manuscript with edits from AS-L, VD, and BM. All authors contributed to the article and approved the submitted version.

## Funding

This work was supported by the United States Geological Survey, Vermont Water Resources and Lake Studies Center 104b grant (subaward award G16AP00087 to L-AG) https://www.uvm.edu/rsenr/vtwatercenter. AS-L is supported by the Agence Nationale de la Recherche (ANR-17-CE13-00001-01 “Amocyst”).

## Conflict of Interest

The authors declare that the research was conducted in the absence of any commercial or financial relationships that could be construed as a potential conflict of interest.

## Publisher’s Note

All claims expressed in this article are solely those of the authors and do not necessarily represent those of their affiliated organizations, or those of the publisher, the editors and the reviewers. Any product that may be evaluated in this article, or claim that may be made by its manufacturer, is not guaranteed or endorsed by the publisher.

## Footnotes

^1^ https://blast.ncbi.nlm.nih.gov/Blast.cgi

^2^ https://www.arb-silva.de/fish-probes/fish-protocols/

^3^ https://www.genome.jp/kegg/

## References

[ref1] Abramoff M. Magalhães P. Ram S. (2004). Image processing with ImageJ. Biophoton. Int. 11, 36–42. doi: 10.1201/978142000561

[ref2] Aguilera A. (2013). Eukaryotic organisms in extreme acidic environments, the Río Tinto case. Life 3, 363–374. doi: 10.3390/life3030363, PMID: 25369810 PMC4187173

[ref3] Alanjary M. Kronmiller B. Adamek M. Blin K. Weber T. Huson D.. (2017). The antibiotic resistant target seeker (ARTS), an exploration engine for antibiotic cluster prioritization and novel drug target discovery. Nucleic Acids Res. 45, W42–W48. doi: 10.1093/nar/gkx360, PMID: 28472505 PMC5570205

[ref4] Al-Khodor S. Price C. T. Kalia A. Kwaik Y. A. (2010). Functional diversity of ankyrin repeats in microbial proteins. Trends Microbiol. 18, 132–139. doi: 10.1016/j.tim.2009.11.004, PMID: 19962898 PMC2834824

[ref5] Altschul S. F. Gish W. Miller W. Myers E. W. Lipman D. J. (1990). Basic local alignment search tool. J. Mol. Biol. 215, 403–410. doi: 10.1016/S0022-2836(05)80360-2 2231712

[ref6] Amaral-Zettler L. (2013). Eukaryotic diversity at pH extremes. Front. Microbiol. 3:441. doi: 10.3389/fmicb.2012.00441 23335919 PMC3547282

[ref7] American Public Health Association (2018). “Standard methods committee of the American Public Health Association, American Water Works Association, and Water Environment Federation. 5310 total organic carbon (TOC).” In Standard Methods for the Examination of Water and Wastewater eds. Lipps W. C. Baxter T. E. Braun-Howland E.. (Washington DC: APHA Press). 10.2105/SMWW.2882.104

[ref8] Andrews S. (2010). FastQC: A Quality Control Tool for High Throughput Sequence Data [Online]. Available at: http://www.bioinformatics.babraham.ac.uk/projects/fastqc/ (Accessed December 9, 2021).

[ref9] Aziz R. K. Bartels D. Best A. A. Dejongh M. Disz T. Edwards R. A.. (2008). The RAST server: rapid annotations using subsystems technology. BMC Genomics 9:75. doi: 10.1186/1471-2164-9-75, PMID: 18261238 PMC2265698

[ref10] Baker B. J. Banfield J. F. (2003). Microbial communities in acid mine drainage. FEMS Microbiol. Ecol. 44, 139–152. doi: 10.1016/S0168-6496(03)00028-X 19719632

[ref11] Baker B. J. Hugenholtz P. Dawson S. C. Banfield J. F. (2003). Extremely acidophilic protists from acid mine drainage host *Rickettsiale*s-lineage endosymbionts that have intervening sequences in their 16S rRNA genes. Appl. Environ. Microbiol. 69, 5512–5518. doi: 10.1128/AEM.69.9.5512-5518.2003, PMID: 12957940 PMC194945

[ref12] Banach A. M. Kuźniar A. Grządziel J. Wolińska A. (2020). *Azolla filiculoides* L. as a source of metal-tolerant microorganisms. PLoS One 15:e0232699. doi: 10.1371/journal.pone.0232699, PMID: 32374760 PMC7202617

[ref13] Bertani B. Ruiz N. (2018). Function and biogenesis of lipopolysaccharides. EcoSal Plus 8. doi: 10.1128/ecosalplus.ESP-0001-2018, PMID: 30066669 PMC6091223

[ref14] Bertelli C. Greub G. (2012). Lateral gene exchanges shape the genomes of amoeba-resisting microorganisms. Front. Cell. Infect. Microbiol. 2:110. doi: 10.3389/fcimb.2012.00110, PMID: 22919697 PMC3423634

[ref15] Bevers L. E. Hagedoorn P.-L. Krijger G. C. Hagen W. R. (2006). Tungsten transport protein A (WtpA) in *Pyrococcus furiosus*: the first member of a new class of tungstate and molybdate transporters. J. Bacteriol. 188, 6498–6505. doi: 10.1128/JB.00548-06, PMID: 16952940 PMC1595483

[ref16] Blin K. Shaw S. Kloosterman A. M. Charlop-Powers Z. Van Wezel G. P. Medema M. H.. (2021). antiSMASH 6.0: improving cluster detection and comparison capabilities. Nucleic Acids Res. 49, W29–W35. doi: 10.1093/nar/gkab335, PMID: 33978755 PMC8262755

[ref17] Brock D. A. Douglas T. E. Queller D. C. Strassmann J. E. (2011). Primitive agriculture in a social amoeba. Nature 469, 393–396. doi: 10.1038/nature09668, PMID: 21248849

[ref18] Buchfink B. Xie C. Huson D. H. (2015). Fast and sensitive protein alignment using DIAMOND. Nat. Methods 12, 59–60. doi: 10.1038/nmeth.3176, PMID: 25402007

[ref19] Chen L.-X. Huang L.-N. Méndez-García C. Kuang J.-L. Hua Z.-S. Liu J.. (2016). Microbial communities, processes and functions in acid mine drainage ecosystems. Curr. Opin. Biotechnol. 38, 150–158. doi: 10.1016/j.copbio.2016.01.013, PMID: 26921733

[ref20] Chen S. Zhou Y. Chen Y. Gu J. (2018). fastp: an ultra-fast all-in-one FASTQ preprocessor. Bioinformatics 34, i884–i890. doi: 10.1093/bioinformatics/bty560, PMID: 30423086 PMC6129281

[ref21] Christie P. J. (2004). Type IV secretion: the Agrobacterium VirB/D4 and related conjugation systems. Biochim. Biophys. Acta, Mol. Cell Res. 1694, 219–234. doi: 10.1016/j.bbamcr.2004.02.013, PMID: 15546668 PMC4845649

[ref22] Clark J. Haskins E. (2014). Sporophore morphology and development in the myxomycetes. Mycosphere 5, 153–170. doi: 10.5943/mycosphere/5/1/7

[ref23] Constancio M. T. L. Sacco L. P. Campanharo J. C. Castellane T. C. L. De Oliveira Souza A. C. Weiss B.. (2020). Exploring the potential of two bacterial consortia to degrade cellulosic biomass for biotechnological applications. Curr. Microbiol. 77, 3114–3124. doi: 10.1007/s00284-020-02136-7, PMID: 32719889

[ref24] Dai D. Okorley B. A. Li Y. Zhang B. (2020). Life cycles of myxogastria *Stemonitopsis typhina* and *Stemonitis fusca* on agar culture. J. Eukaryot. Microbiol. 67, 66–75. doi: 10.1111/jeu.12754, PMID: 31408563 PMC6973090

[ref26] Dean A. P. Lynch S. Rowland P. Toft B. D. Pittman J. K. White K. N. (2013). Natural wetlands are efficient at providing long-term metal remediation of freshwater systems polluted by acid mine drainage. Environ. Sci. Technol. 47, 12029–12036. doi: 10.1021/es4025904, PMID: 24088022

[ref25] De Gonzalo G. Colpa D. I. Habib M. H. M. Fraaije M. W. (2016). Bacterial enzymes involved in lignin degradation. J. Bacteriol. 236, 110–119. doi: 10.1016/j.jbiotec.2016.08.011 27544286

[ref27] Desiderato J. G. Alvarenga D. O. Constancio M. T. Alves L. Varani A. M. (2018). The genome sequence of *Dyella jiangningensis* FCAV SCS01 from a lignocellulose-decomposing microbial consortium metagenome reveals potential for biotechnological applications. Genet. Mol. Biol. 41, 507–513. doi: 10.1590/1678-4685-gmb-2017-0155, PMID: 29767666 PMC6082245

[ref28] Dunn J. D. Bosmani C. Barisch C. Raykov L. Lefrançois L. H. Cardenal-Muñoz E.. (2018). Eat prey, live: *Dictyostelium discoideum* as a model for cell-autonomous defenses. Front. Immunol. 8:1906. doi: 10.3389/fimmu.2017.01906, PMID: 29354124 PMC5758549

[ref01] Edgar R. C. (2004). MUSCLE: multiple sequence alignment with high accuracy and high throughput. Nucleic Acids Res. 32 1792–1797. 15034147 10.1093/nar/gkh340 PMC390337

[ref29] Fisher R. M. Henry L. M. Cornwallis C. K. Kiers E. T. West S. A. (2017). The evolution of host-symbiont dependence. Nat. Commun. 8:15973. doi: 10.1038/ncomms15973, PMID: 28675159 PMC5500886

[ref30] Gao Y. Tao W. Yan S.-Z. Chen S.-L. (2017). The life cycle of *Didymium laxifilum* and *Physarum album* on oat agar culture. J. Eukaryot. 64, 457–463. doi: 10.1111/jeu.12383 27862633

[ref31] Gast R. J. Sanders R. W. Caron D. A. (2009). Ecological strategies of protists and their symbiotic relationships with prokaryotic microbes. Trends Microbiol. 17, 563–569. doi: 10.1016/j.tim.2009.09.001, PMID: 19828317

[ref32] Giddings L.-A. Chlipala G. Driscoll H. Bhave K. Kunstman K. Green S.. (2020a). Seasonal Ely Copper Mine Superfund site shotgun metagenomic and metatranscriptomic data analysis. Data Brief 32:106282. doi: 10.1016/j.dib.2020.106282, PMID: 32984474 PMC7494679

[ref33] Giddings L.-A. Chlipala G. Kunstman K. Green S. Morillo K. Bhave K.. (2020b). Characterization of an acid rock drainage microbiome and transcriptome at the Ely Copper Mine Superfund site. PLoS One 15:e0237599. doi: 10.1371/journal.pone.0237599, PMID: 32785287 PMC7423320

[ref34] Görtz H.-D. (2006). “Symbiotic associations between ciliates and prokaryotes,” in The Prokaryotes. eds. Dworkin M. Falkow S. Rosenberg E. Schleifer K. H. Stackebrandt E. (New York, NY: Springer), 364–402.

[ref35] Greub G. Raoult D. (2004). Microorganisms resistant to free-living amoebae. Clin. Microbiol. Rev. 17, 413–433. doi: 10.1128/CMR.17.2.413-433.2004, PMID: 15084508 PMC387402

[ref36] Gruzdev E. V. Beletsky A. V. Kadnikov V. V. Mardanov A. V. Ivanov M. V. Karnachuk O. V.. (2020). Diversity of eukaryotic microorganisms in the drainage waters of a coal open-cast mine. Microbiology 89, 641–646. doi: 10.1134/S0026261720050100

[ref37] Hadziavdic K. Lekang K. Lanzen A. Jonassen I. Thompson E. M. Troedsson C. (2014). Characterization of the 18S rRNA gene for designing universal eukaryote specific primers. PLoS One 9:e87624. doi: 10.1371/journal.pone.0087624, PMID: 24516555 PMC3917833

[ref38] He L. Xiafang S. Qi W. Li X. (2017). Plant Growth Promoting Bacterium for Reducing Heavy Metal Content in Leaf Vegetables and Application Thereof China Patent Application. CN201710938596.3A.

[ref39] Hilbi H. Hoffmann C. Harrison C. F. (2011). *Legionella* spp. outdoors: colonization, communication and persistence. Environ. Microbiol. Rep. 3, 286–296. doi: 10.1111/j.1758-2229.2011.00247.x, PMID: 23761274

[ref40] Hoque M. M. Noorian P. Espinoza-Vergara G. Manuneedhi Cholan P. Kim M. Rahman M. H.. (2021). Adaptation to an amoeba host drives selection of virulence-associated traits in *Vibrio cholerae*. ISME J. 16, 856–867. doi: 10.1038/s41396-021-01134-2, PMID: 34654895 PMC8857207

[ref41] Janusz G. Pawlik A. Sulej J. Świderska-Burek U. Jarosz-Wilkołazka A. Paszczyński A. (2017). Lignin degradation: microorganisms, enzymes involved, genomes analysis and evolution. FEMS Microbiol. Rev. 41, 941–962. doi: 10.1093/femsre/fux049, PMID: 29088355 PMC5812493

[ref42] Johnson D. B. Aguilera A. (2015). “The microbiology of extremely acidic environments,” in Manual of environmental microbiology, *4th Edn*, eds. Yates M. V. Cindy H. Nakatsu C. H. Miller R. V. Pillai S. D. (New York, NY: ASM Press). doi: 10.1128/9781555818821.ch4.3.1

[ref43] Johnson D. B. Rang L. (1993). Effects of acidophilic protozoa on populations of metal-mobilizing bacteria during the leaching of pyritic coal. Microbiology 139, 1417–1423.

[ref44] Kalyaanamoorthy S. Minh B. Q. Wong T. K. F. Von Haeseler A. Jermiin L. S. (2017). ModelFinder: fast model selection for accurate phylogenetic estimates. Nat. Methods 14, 587–589. doi: 10.1038/nmeth.4285, PMID: 28481363 PMC5453245

[ref45] Kalyanasundaram I. (2004). A positive ecological role for tropical Myxomycetes in association with bacteria. Syst. Geogr. Plants 74, 239–242. doi: 10.2307/3668492

[ref46] Kamimura N. Takahashi K. Mori K. Araki T. Fujita M. Higuchi Y.. (2017). Bacterial catabolism of lignin-derived aromatics: new findings in a recent decade: update on bacterial lignin catabolism. Environ. Microbiol. Rep. 9, 679–705. doi: 10.1111/1758-2229.12597, PMID: 29052962

[ref47] Kanehisa M. Goto S. (2000). KEGG: Kyoto encyclopedia of genes and genomes. Nucleic Acids Res. 28, 27–30. doi: 10.1093/nar/28.1.27, PMID: 10592173 PMC102409

[ref48] Kefeni K. K. Msagati T. A. Mamba B. B. (2017). Acid mine drainage: prevention, treatment options, and resource recovery: a review. J. Clean. Prod. 151, 475–493. doi: 10.1016/j.jclepro.2017.03.082

[ref49] Kolmogorov M. Bickhart D. M. Behsaz B. Gurevich A. Rayko M. Shin S. B.. (2020). metaFlye: scalable long-read metagenome assembly using repeat graphs. Nat. Methods 17, 1103–1110. doi: 10.1038/s41592-020-00971-x, PMID: 33020656 PMC10699202

[ref50] Kolmogorov M. Yuan J. Lin Y. Pevzner P. A. (2019). Assembly of long, error-prone reads using repeat graphs. Nat. Biotechnol. 37, 540–546. doi: 10.1038/s41587-019-0072-8, PMID: 30936562

[ref51] Koren S. Walenz B. P. Berlin K. Miller J. R. Bergman N. H. Phillippy A. M. (2017). Canu: scalable and accurate long-read assembly via adaptive k-mer weighting and repeat separation. Genome Res. 27, 722–736. doi: 10.1101/gr.215087.116, PMID: 28298431 PMC5411767

[ref52] Krashevska V. Malysheva E. Klarner B. Mazei Y. Maraun M. Widyastuti R.. (2018). Micro-decomposer communities and decomposition processes in tropical lowlands as affected by land use and litter type. Oecologia 187, 255–266. doi: 10.1007/s00442-018-4103-9, PMID: 29497833

[ref53] Krashevska V. Sandmann D. Marian F. Maraun M. Scheu S. (2017). Leaf litter chemistry drives the structure and composition of soil testate amoeba communities in a tropical montane rainforest of the Ecuadorian Andes. Microb. Ecol. 74, 681–690. doi: 10.1007/s00248-017-0980-4, PMID: 28389728

[ref54] Kutschera U. Hoppe T. (2019). Plasmodial slime molds and the evolution of microbial husbandry. Theory Biosci. 138, 127–132. doi: 10.1007/s12064-019-00285-3, PMID: 30809766

[ref55] Langmead B. Salzberg S. L. (2012). Fast gapped-read alignment with bowtie 2. Nat. Methods 9, 357–359. doi: 10.1038/nmeth.1923, PMID: 22388286 PMC3322381

[ref56] Leger A. Leonardi T. (2019). pycoQC, interactive quality control for Oxford Nanopore sequencing. J. Open Source Softw. 4:1236. doi: 10.21105/joss.01236

[ref57] Manni M. Berkeley M. R. Seppey M. Simão F. A. Zdobnov E. M. (2021). BUSCO update: novel and streamlined workflows along with broader and deeper phylogenetic coverage for scoring of eukaryotic, prokaryotic, and viral genomes. Mol. Biol. Evol. 38, 4647–4654. doi: 10.1093/molbev/msab199, PMID: 34320186 PMC8476166

[ref58] Marchler-Bauer A. Bryant S. H. (2004). CD-search: protein domain annotations on the fly. Nucleic Acids Res. 32, W327–W331. doi: 10.1093/nar/gkh454, PMID: 15215404 PMC441592

[ref59] McCutcheon J. P. Moran N. A. (2012). Extreme genome reduction in symbiotic bacteria. Nat. Rev. Microbiol. 10, 13–26. doi: 10.1038/nrmicro2670, PMID: 22064560

[ref60] McGee C. Storey S. Clipson N. Doyle E. (2017). Soil microbial community responses to contamination with silver, aluminium oxide and silicon dioxide nanoparticles. Ecotoxicology 26, 449–458. doi: 10.1007/s10646-017-1776-5, PMID: 28197855

[ref61] Méndez-García C. Peláez A. I. Mesa V. Sánchez J. Golyshina O. V. Ferrer M. (2015). Microbial diversity and metabolic networks in acid mine drainage habitats. Front. Microbiol. 6:475. doi: 10.3389/fmicb.2015.00475 26074887 PMC4448039

[ref62] Mills S. D. Lim C.-K. Cooksey D. A. (1994). Purification and characterization of CopR, a transcriptional activator protein that binds to a conserved domain (cop box) in copper- inducible promoters of *Pseudomonas* syringae. Mol. Gen. Genet. 244, 341–351. doi: 10.1007/BF00286685, PMID: 8078459

[ref63] Minh B. Q. Schmidt H. A. Chernomor O. Schrempf D. Woodhams M. D. Von Haeseler A.. (2020). IQ-TREE 2: new models and efficient methods for phylogenetic inference in the genomic era. Mol. Biol. Evol. 37, 1530–1534. doi: 10.1093/molbev/msaa015, PMID: 32011700 PMC7182206

[ref64] Moliner C. Fournier P.-E. Raoult D. (2010). Genome analysis of microorganisms living in amoebae reveals a melting pot of evolution. FEMS Microbiol. Rev. 34, 281–294. doi: 10.1111/j.1574-6976.2009.00209.x, PMID: 20132312

[ref65] Mungan M. D. Alanjary M. Blin K. Weber T. Medema M. H. Ziemert N. (2020). ARTS 2.0: feature updates and expansion of the antibiotic resistant target seeker for comparative genome mining. Nucleic Acids Res. 48, W546–W552. doi: 10.1093/nar/gkaa374, PMID: 32427317 PMC7319560

[ref66] Naushad S. Adeolu M. Wong S. Sohail M. Schellhorn H. E. Gupta R. S. (2015). A phylogenomic and molecular marker based taxonomic framework for the order Xanthomonadales: proposal to transfer the families *Algiphilaceae* and *Solimonadaceae* to the order Nevskiales ord. nov. and to create a new family within the order Xanthomonadales, the family *Rhodanobacteraceae* fam. nov., containing the genus *Rhodanobacter* and its closest relatives. Antonie Van Leeuwenhoek 107, 467–485. doi: 10.1007/s10482-014-0344-8, PMID: 25481407

[ref67] Nordstrom D. K. Blowes D. W. Ptacek C. J. (2015). Hydrogeochemistry and microbiology of mine drainage: an update. J. Appl. Geochem. 57, 3–16. doi: 10.1016/j.apgeochem.2015.02.008

[ref68] Ohkuma M. Noda S. Hattori S. Iida T. Yuki M. Starns D.. (2015). Acetogenesis from H_2_ plus CO_2_ and nitrogen fixation by an endosymbiotic spirochete of a termite-gut cellulolytic protist. Proc. Natl. Acad. Sci. U. S. A. 112, 10224–10230. doi: 10.1073/pnas.1423979112 25979941 PMC4547241

[ref69] Ou F.-H. Gao Z.-H. Chen M.-H. Bi J.-Y. Qiu L.-H. (2019). *Dyella dinghuensis* sp. nov. and *Dyella choica* sp. nov., isolated from forest soil. Int. J. Syst. Evol. Microbiol. 69, 1496–1503. doi: 10.1099/ijsem.0.003356, PMID: 30900974

[ref70] Oxford Nanopore Technologies (2018). Medaka v.0.8.1 [Online]. Available at: https://github.com/nanoporetech/medaka (Accessed December 9, 2021).

[ref71] Pal C. Bengtsson-Palme J. Rensing C. Kristiansson E. Larsson D. G. J. (2013). BacMet: antibacterial biocide and metal resistance genes database. Nucleic Acids Res. 42, D737–D743. doi: 10.1093/nar/gkt1252 24304895 PMC3965030

[ref72] Park I. Tabelin C. B. Jeon S. Li X. Seno K. Ito M.. (2019). A review of recent strategies for acid mine drainage prevention and mine tailings recycling. Chemosphere 219, 588–606. doi: 10.1016/j.chemosphere.2018.11.053, PMID: 30554047

[ref73] Parks D. H. Imelfort M. Skennerton C. T. Hugenholtz P. Tyson G. W. (2015). CheckM: assessing the quality of microbial genomes recovered from isolates, single cells, and metagenomes. Genome Res. 25, 1043–1055. doi: 10.1101/gr.186072.114, PMID: 25977477 PMC4484387

[ref74] Perron K. Caille O. Rossier C. Van Delden C. Dumas J.-L. Köhler T. (2004). CzcR-CzcS, a two-component system involved in heavy metal and carbapenem resistance in *Pseudomonas aeruginosa*. J. Biol. Chem. 279, 8761–8768. doi: 10.1074/jbc.M312080200, PMID: 14679195

[ref75] Pfaff J. D. (1993). Method 300.0 Determination of Inorganic Anions by Ion Chromatography. US Environmental Protection Agency, Office of Research and Development, Environmental Monitoring Systems Laboratory 28.

[ref76] Pujalte M. J. Lucena T. Ruvira M. A. Arahal D. R. Macián M. C. (2014). “The family *Rhodobacteraceae*,” in The Prokaryotes: Alphaproteobacteria and Betaproteobacteria. eds. Rosenberg E. Delong E. F. Lory S. Stackebrandt E. Thompson F. (Berlin, Heidelberg: Springer Berlin Heidelberg), 439–512.

[ref77] Rambaut A. (2021). Fig Tree v. 1.4.4. 2018. [Online]. Available at: http://tree.bio.ed.ac.uk/software/figtree/ (Accessed December 9, 2021).

[ref78] Rezaie B. Anderson A. (2020). Sustainable resolutions for environmental threat of the acid mine drainage. Sci. Total Environ. 717:137211. doi: 10.1016/j.scitotenv.2020.137211, PMID: 32062234

[ref79] Samba-Louaka A. Delafont V. Rodier M.-H. Cateau E. Héchard Y. (2019). Free-living amoebae and squatters in the wild: ecological and molecular features. FEMS Microbiol. Rev. 43, 415–434. doi: 10.1093/femsre/fuz011, PMID: 31049565

[ref80] Sánchez-Andrea I. Sanz J. L. Bijmans M. F. M. Stams A. J. M. (2014). Sulfate reduction at low pH to remediate acid mine drainage. J. Hazard. Mater. 269, 98–109. doi: 10.1016/j.jhazmat.2013.12.032, PMID: 24444599

[ref81] Schulz-Bohm K. Geisen S. Wubs E. R. J. Song C. De Boer W. Garbeva P. (2017). The prey’s scent – volatile organic compound mediated interactions between soil bacteria and their protist predators. ISME J. 11, 817–820. doi: 10.1038/ismej.2016.144, PMID: 27911440 PMC5322296

[ref82] Seemann T. (2014). Prokka: rapid prokaryotic genome annotation. Bioinformatics 30, 2068–2069. doi: 10.1093/bioinformatics/btu153, PMID: 24642063

[ref83] Shi Y. Queller D. C. Tian Y. Zhang S. Yan Q. He Z.. (2021). The ecology and evolution of amoeba-bacterium interactions. Appl. Environ. Microbiol. 87, e01866–e01820. doi: 10.1128/AEM.01866-20 33158887 PMC7783332

[ref84] Sirim D. Wagner F. Wang L. Schmid R. D. Pleiss J. (2011). The Laccase engineering database: a classification and analysis system for laccases and related multicopper oxidases. Database 2011:bar006. doi: 10.1093/database/bar006 21498547 PMC3077825

[ref85] Stephenson S.L. Stempen H. (1994). Myxomycetes: A Handbook of Slime Molds. Portland, Oregon: Timber Press.

[ref86] Strassmann J. E. Shu L. (2017). Ancient bacteria–amoeba relationships and pathogenic animal bacteria. PLoS Biol. 15:e2002460. doi: 10.1371/journal.pbio.2002460, PMID: 28463965 PMC5412987

[ref87] United States Environmental Protection Agency (2003). Contaminant Candidate List Regulatory Determination Support Document for Sulfate EPA 815-R-03-016. United States Environmental Protection Agency.

[ref88] Valentín-Vargas A. Neilson J. W. Root R. A. Chorover J. Maier R. M. (2018). Treatment impacts on temporal microbial community dynamics during phytostabilization of acid-generating mine tailings in semiarid regions. Sci. Total Environ. 618, 357–368. doi: 10.1016/j.scitotenv.2017.11.010, PMID: 29132003 PMC5773348

[ref89] Valkanas M. M. Rosso T. Packard J. E. Trun N. J. (2021). Limited carbon sources prevent sulfate remediation in circumneutral abandoned mine drainage. FEMS Microbiol. Ecol. 97:fiaa262. doi: 10.1093/femsec/fiaa262 33417684

[ref90] Weon H.-Y. Anandham R. Kim B.-Y. Hong S.-B. Jeon Y.-A. Kwon S.-W. (2009). *Dyella soli* sp. nov. and *Dyella terrae* sp. nov., isolated from soil. Int. J. Syst. Evol. Microbiol. 59, 1685–1690. doi: 10.1099/ijs.0.004838-0, PMID: 19542132

[ref91] Wheeler T. J. Eddy S. R. (2013). nhmmer: DNA homology search with profile HMMs. Bioinformatics 29, 2487–2489. doi: 10.1093/bioinformatics/btt403, PMID: 23842809 PMC3777106

[ref92] Wick R. R. Holt K. E. (2021). Polypolish: short-read polishing of long-read bacterial genome assemblies. bioRxiv [Preprint]. 10.1371/journal.pcbi.1009802 PMC8812927 35073327

[ref93] Wright E. (2013). DECIPHER: Database Enabled Code for Ideal Probe Hybridization Employing R. R package version 1.10.0. http://www.bioconductor.org/packages/release/bioc/html/DECIPHER.html.

[ref94] Xie C.-H. Yokota A. (2005). *Dyella japonica* gen. nov., sp. nov., a γ-proteobacterium isolated from soil. Int. J. Syst. Evol. Microbiol. 55, 753–756. doi: 10.1099/ijs.0.63377-0, PMID: 15774657

[ref95] Yilmaz L. S. Parnerkar S. Noguera D. R. (2011). mathFISH, a web tool that uses thermodynamics-based mathematical models for *in silico* evaluation of oligonucleotide probes for fluorescence *in situ* hybridization. Appl. Environ. Microbiol. 77, 1118–1122. doi: 10.1128/AEM.01733-10, PMID: 21148691 PMC3028703

[ref96] Yoon S.-H. Ha S.-M. Kwon S. Lim J. Kim Y. Seo H.. (2017). Introducing EzBioCloud: a taxonomically united database of 16S rRNA gene sequences and whole-genome assemblies. Int. J. Syst. Evol. Microbiol. 67, 1613–1617. doi: 10.1099/ijsem.0.001755, PMID: 28005526 PMC5563544

[ref97] Zhao F. Guo X.-Q. Wang P. He L.-Y. Huang Z. Sheng X.-F. (2013). *Dyella jiangningensis* sp. nov., a γ-proteobacterium isolated from the surface of potassium-bearing rock. Int. J. Syst. Evol. Microbiol. 63, 3154–3157. doi: 10.1099/ijs.0.048470-0, PMID: 23435246

[ref98] Zhu X. Moodley O. Wang Q. Li Y. (2019). The life cycles of two species of Myxomycetes in Physarales, *Physarum rigidum* and *Didymium squamulosum*. J. Basic Microbiol. 59, 658–664. doi: 10.1002/jobm.201800594, PMID: 30900739

