# Supplementary material for: Isolation and Genome Analysis of an Amoeba-Associated Bacterium Dyella terrae Strain Ely Copper Mine From Acid Rock Drainage in Vermont, United States
Source: Front Microbiol. 2022 May 23;13:856908. doi: 10.3389/fmicb.2022.856908 (PMC9169046; doi:10.3389/fmicb.2022.856908)
Supplement: Supplementary file 4 [file Data_Sheet_1.docx]

Supplementary Material

Isolation and genome analysis of an amoeba-associated bacterium *Dyella terrae* Ely Copper Mine from acid rock drainage at Ely Copper Mine in Vermont, USA

Lesley-Ann Giddings^1,2*^, Kevin Kunstman^3^, Bouziane Moumen^4^, Laurent Asiama^2^, Stefan Green^3^, Vincent Delafont^4^, Matthew Brockley^2^, Ascel Samba-Louaka^4^

Affiliations:

1. Department of Chemistry, Smith College, Northampton, MA, 01063
2. Department of Chemistry & Biochemistry, Middlebury College, Middlebury, VT, 05753, USA
3. Research Resources Center, University of Illinois at Chicago, Chicago, IL, 60612, USA
4. Laboratoire Ecologie et Biologie des Interactions, Université de Poitiers, Poitiers, France

**ICAP-MS analysis of sediment and water**

A Thermofisher inductively-coupled argon plasma mass spectrometry (ICAP-MS) was used to test water samples for dissolved and total trace metal concentrations using internal and external standards. The instrument was tuned using the THERMO-5A (Inorganic Ventures, VA) multielement standard according to the manufacturer’s procedures. Performance was verified using the THERMO-4AREV multielement standard to ensure instrument stability (<5%). Helium was used as a collision gas at a rate of 4.9 ml/min. The following elements had dwell times of 0.01 s: Sc, Y, In, Sn, Ba, Tb, and Bi. All other detected elements had dwell times of 0.05 s. The estimated total run time for each sample was 13s 800 ms.

The 2008ISS multielement standard (Inorganic Ventures; Christiansburg, VA) was used as an internal reference for all samples at a concentration of 100 ppb to correct for instrument drift. All samples including the standards were spiked with the internal standard. See Supplemental Table 1 for a list of all reference and internal standards used in ICAP-MS analyses. The following five standards were used: 1) a 5% nitric acid solution containing 10.1 ppm Na, Mg, Ca, K, Al, Si, and Fe as well as 100 ppb IV71A, IV71B, and 2008ISS (Sc, Y, Bi, In, and Tb); 2) a 5% nitric acid solution containing 5.05 ppm Na, Mg, Ca, K, Al, Si, and Fe, 50 ppb IV71A and IV71B, and 100 ppb 2008ISS (Sc, Y, Bi, In, and Tb); 3) a 5% nitric acid solution containing 1.01 ppm Na, Ms, Ca, K, Al, Si, and Fe, 10 ppb IV71A and IV1B, and 100 ppb 2008ISS (Sc, Y, Bi, In, and Tb); 4) a 5% nitric acid solution containing 101 ppb Na, Mg, Ca, K, Al, Si, and Fe, 1 ppb IV71A and IV71B, and 100 ppb 2008ISS (Sc, Y, Bi, In, and Tb); and 5) a 5% nitric acid solution containing 100 ppb 2008ISS (Sc, Y, Bi, In, and Tb). The NIST standard reference material 1643f was used to ensure that the instrument was calibrated within 15% per element. We report only estimated values for Ca and Al (see Supplemental Table 2) due to the 1) Ca level in 1643f being three times more concentrated than the most concentrated standard used to make the standard curves but the value was within 15% of the reported Ca concentration in 1643; and 2) trace amounts of Al contaminating standards containing low concentrations of Al precluding our verification of being within 15% of the 1643f reference, which also contained low concentrations of Al, but our actual samples had higher concentrations of aluminum and should be less affected by this. All standard curves had R^2^ values of 0.99.

| Standard reference material | Sample matrix | Certified values of elements |
| --- | --- | --- |
| Traces (1:1 mixture of IV-ICPMS-71A and IV-ICPMS-71B from Inorganic Ventures; Christiansburg, VA) | sediment | 1 ppm Rd, In, and Bi |
| 2008ISS (Inorganic Ventures; Christiansburg, VA) | water | Bi (20.01 ± 0.11 ppb), Sc (20.01 ± 0.13 ppb), Y (20.01 ± 0.08 ppb) |
| 1643f (NIST; Gaithersburg, MD) | water | Al (133.8 ± 1.2 ppm), Sb (55.45 ± 0.40 ppm), As (57.42 ± 0.38 ppm), Ba (518.2 ± 7.3 ppm), Be (13.67 ± 0.12 ppm), Bi (12.62 ± 0.11 ppm), B (152.3 ± 6.6 ppm), Cd (5.89 ± 0.13 ppm), Ca (29,430 ± 330 ppm), Cr (18.50 ± 0.10 ppm), Co (25.30 ± 0.17 ppm), Cu (21.66 ± 0.71 ppm), Fe (193.44 ± 0.78 ppm), Pb (18.488 ± 0.084 ppm), Li (16.59 ± 0.35 ppm), Mg (7,454 ± 60 ppm), Mn (37.14 ± 0.60 ppm), Mo (115.3 ± 1.7 ppm), Ni (59.8 ± 1.4 ppm), K (1,932.6 ± 9.4 ppm), Rb (12.64 ± 0.13 ppm), Se (11.700 ± 0.081 ppm), Ag (0.9703 ± 0.0055 ppm), Na (18,830 ± 250 ppm), Sr (314 ± 19 ppm), Te (0.9770 ± 0.0084 ppm), Tl (6.892 ± 0.035 ppm), V (36.07 ± 0.28 ppm), Zn (74.4 ± 1.7 ppm) |

**Supplementary Table 1.** References used to analyze water or sediment by ICAP-MS.

|  | November 2018 (mg/L) |
| --- | --- |
| Dissolved metal | 12.4 Ca, 4.14 Al |
| Total metal | 15.3 Ca, 5.29 Al |

**Supplementary Table 2**. Estimate concentrations of calcium and aluminum ions in water samples.

**Supplementary Figure 1**. **Maximum likelihood tree based on near complete 18S rRNA gene sequences showing the phylogeny the *Stemonitis* sp. strain Ely Copper Mine, within the Stemonitida group.** Sequences of the sister group Physarida were used as an outgroup. Bootstrap (left) and SH-like approximate likelihood ratio (right) support are expressed at nodes, in percentage. Bar represents 0.1 substitutions per nucleotide position. To determine the phylogenetic position of the *Stemonitis* isolate, a representative set of 18S rRNA gene sequences were compiled from the non-redundant nucleotide database of the NCBI. A total of 38 full or near-full length sequences, including the complete 18S rRNA sequence for the amoeba were collected, manually inspected, and aligned using MUSCLE v3.8.31(Edgar, 2004), resulting in a global alignment of 5451 positions, including 1109 informative nucleotide sites. ModelFinder (Kalyaanamoorthy et al., 2017) was used to determine the best substitution model for phylogenetic inferences. Based on the best Bayesian information criterion score, the TIM2+F+I+G4 model was selected. Inference of the phylogeny was done by maximum likelihood using IQ-TREE v 2.0.3 (Minh et al., 2020). The robustness of the inference was tested by further applying 1000 iterations of conventional bootstraps and SH-approximate likelihood ratio tests.

| KEGG annotated *Dyella terrae* species Ely Copper Mine gene | KEGG annotated *Dyella terrae* KACC 12748 gene | Gene name | Assigned KOs |
| --- | --- | --- | --- |
| DYST_00031 bifunctional sulfate adenylyltransferase/adenylylsulfate kinase | lcl\|SIZZ01000001.1_prot_TBR39240.1_612 bifunctional sulfate adenylyltransferase/adenylylsulfate kinase | *sat* | K00958 |
| DYST_02109 sulfate adenylyltransferase subunit CysN | lcl\|SIZZ01000001.1_prot_TBR40342.1_1831 [gene=*cysC*] adenylyl-sulfate kinase | *cysNC* | K00955 |
| DYST_02110 sulfate adenylyltransferase subunit CysD | lcl\|SIZZ01000001.1_prot_TBR40343.1_1832 sulfate adenylyltransferase subunit | *cysD* | K00957 |
| DYST_00030 phosphoadenylyl-sulfate reductase | lcl\|SIZZ01000001.1_prot_TBR39239.1_611 phosphoadenylyl-sulfate reductase | *cysH* | K00390 |
| DYST_00029 assimilatory sulfite reductase (NADPH) hemoprotein subunit | lcl\|SIZZ01000001.1_prot_TBR39238.1_610 assimilatory sulfite reductase (NADPH) hemoprotein subunit | *cysI* | K00381 |
| DYST_00028 assimilatory sulfite reductase (NADPH) flavoprotein subunit; DYST_01808 sulfite reductase subunit alpha | lcl\|SIZZ01000001.1_prot_TBR39237.1_609 assimilatory sulfite reductase (NADPH) flavoprotein subunit; lcl\|SIZZ01000001.1_prot_TBR40028.1_1481 sulfite reductase flavoprotein subunit alpha | *cysJ* | K00380 |

**Supplementary Table 3.** Unique *D. terrae* strain Ely Copper Mine and *D. terrae* KACC 12748 genes involved in sulfate reduction based on KO annotations using the KEGG automatic annotation server. Gene identifications for multiple KOs are included.

| BRITE Level 1 Observations | Number of assigned KOs to *D. terrae* species Ely Copper Mine | Number of assigned KOs to *D. terrae* KACC 12748 |
| --- | --- | --- |
| Metabolism | 505 | 625 |
| Genetic Information Processing | 49 | 167 |
| Environmental Information Processing | 233 | 174 |
| Cellular Processes | 202 | 129 |
| Organismal Systems | 64 | 39 |
| Human Diseases | 103 | 86 |
| BRITE Hierarchies | 395 | 961 |
| Not Included in the BRITE Hierarchy | 309 | 234 |

**Supplementary Table 4.** The number of unique *D. terrae* species Ely Copper Mine and *D. terrae* KACC 12748 genes annotated at BRITE level 1 using the KEGG automatic annotation server. This summarizes KEGG supplemental file where level 1 is based on A groupings. Duplicated KOs are not included.

**Supplementary Table 5.** See supplementary material file (Table 2.XLSX) for KO and level 1 BRITE annotations for the *D. terrae* species Ely Copper Mine genome.

| KEGG annotated *Dyella terrae* species Ely Copper Mine gene | KEGG annotated *Dyella terrae* KACC 12748 gene | Gene name | Assigned KOs |
| --- | --- | --- | --- |
| DYST_03873 | lcl\|SIZZ01000002.1_prot_TBR36963.1_2715 | VirB4 | K03199 |
| DYST_03881 | lcl\|SIZZ01000002.1_prot_TBR36969.1_2722 | VirB8 | K03203 |
| DYST_03880 | lcl\|SIZZ01000002.1_prot_TBR36968.1_2721 | VirB9 | K03204 |
| DYST_03879 | lcl\|SIZZ01000002.1_prot_TBR37323.1_2720 | VirB10 | K03195 |
| DYST_03878 | lcl\|SIZZ01000002.1_prot_TBR36967.1_2719 | VirB11 | K03196 |
| DYST_03865,DYST_00180 | lcl\|SIZZ01000002.1_prot_TBR36960.1_2712 | VirD4 | K03205 |
| DYST_04181 | lcl\|SIZZ01000001.1_prot_TBR40282.1_1767 | DsbC | K03981 |
| DYST_00178 | lcl\|SIZZ01000002.1_prot_TBR36908.1_2657 | TrbB | K20527 |
| DYST_00177 |  | TrbC | K20528 |
| DYST_00176 |  | TrbD | K20529 |
| DYST_00175 | lcl\|SIZZ01000002.1_prot_TBR37320.1_2660 | 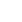  \| TrbE \| \| --- \| | K20530 |
| DYST_00171 | lcl\|SIZZ01000002.1_prot_TBR36911.1_2661 | TrbF | K20531 |
| DYST_00170 | lcl\|SIZZ01000002.1_prot_TBR36912.1_2662 | 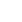  \| TrbG \| \| --- \| | K20532 |
| DYST_00169 | lcl\|SIZZ01000002.1_prot_TBR36914.1_2664 | TrbI | K20533 |
| DYST_00174 |  | 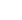  \| TrbJ \| \| --- \| | K20266 |
| DYST_00172 |  | TrbL | K07344 |

**Supplementary Table 6.** Unique *D. terrae* strain Ely Copper Mine and *D. terrae* KACC 12748 genes involved type IV secretion systems based on KO annotations using the KEGG automatic annotation server. Gene identifications for multiple KOs are included.

**Supplementary Table 7.** See supplementary material file (Table 3.XLSX) for KO and level 1 BRITE annotations for the *D. terrae* KACC 12748 genome.

| *Enzymes* | *EC number* | *D. terrae Ely Copper Mine* | *D. terrae*  KACC 12748 | *D. jiangningensis FCAV SCS01* | *D. jiangningensis SBZ 3-12* |
| --- | --- | --- | --- | --- | --- |
| *(****Hemi)cellulose degradation*** | | | | | |
| *Alpha-amylase* | *3.2.1.1* | 1 | 1 | 1 | 1 |
| *Alpha-galactosidase* | *3.2.1.22* | 1 | *n.d.* | 1 | 1 |
| *Alpha-galactosidase precursor* | *3.2.1.22* | *n.d.* | *n.d.* | *n.d.* | *n.d.* |
| *Alpha-glucosidase* | *3.2.1.20* | 1 | *n.d.* | 1 | *n.d.* |
| *Alpha-glucuronidase* | *3.2.1.139* | *n.d.* | *n.d.* | *n.d.* | *n.d.* |
| *Alpha-L-fucosidase* | *3.2.1.51* | 2 | *n.d.* | 3 | 3 |
| *Alpha-N-acetylglucosaminidase* | *3.2.1.50* | 1 | *n.d.* | 1 | 1 |
| *Alpha-1,2-mannosidase* | *3.2.1.24* | 4 | 2 | 6 | 7 |
| *Alpha-xylosidase* | *3.2.1.177* | 2 | 1 | 1 | 1 |
| *Beta-galactosidase* | *3.2.1.23* | 3 | *n.d.* | 3 | 2 |
| *Beta-mannosidase* | *3.2.1.25* | 1 | *n.d.* | 1 | 2 |
| *Xylan 1,4-beta-xylosidase* | *3.2.1.37* | 2 | 1 | 2 | 2 |
| *Endoglucanase* | *3.2.1.4* | *n.d.* | *n.d.* | *n.d.* | *n.d.* |
| *Glucoamylase* | *3.2.1.3* | 1 | 1 | 1 | 1 |
| *Trehalase* | *3.2.1.28* | 1 | *n.d.* | 1 | 1 |
| *Malto-oligosyltrehalose trehalohydrolase* | *3.2.1.141* | *n.d.* | *n.d.* | 1 | 1 |
| *Maltodextrin glucosidase* | *3.2.1.20* | 2 | 2 | 2 | 1 |
| *Alpha-N-acetylgalactosaminidase* | *3.2.1.49* | 1 | 1 | 1 | 1 |
| *Xylanase* | *3.2.1.8* | 1 | 1 | 1 | 1 |
| *Endo-1,4-beta-xylanase A precursor* | *3.2.1.8* | *n.d.* | *n.d.* | *n.d.* | *n.d.* |
| ***Chitin degradation*** | | | | | |
| *Beta-hexosaminidase* | *3.2.1.52* | *n.d.* | *n.d.* | *n.d.* | *n.d.* |
| *Chitinase* | *3.2.1.14* | 2 | 1 | 2 | 3 |
| *Total glycoside hydrolases* |  | 26 | 12 | 29 | 29 |

**Supplementary Table 8.** Comparison of lignocellulose decomposition genes among RAST-annotated *Dyella* genomes. *D. terrae* KACC 12748 (BioProject: PRJNA523522), *D. jiangningensis FCAV SCS01* (BioProject: PRJNA386033), and *D. jiangningensis SBZ 3-12* (BioProject: PRJEB16000). The RastTk annotation pipeline was used. (n.d. = not detected).

| gene name | query_name | sequence_evalue | sequence_score | best_domain_evalue | description |
| --- | --- | --- | --- | --- | --- |
| ***D. terrae* strain Ely Copper Mine** | | | | | |
| DYST_01432 | lcced.SFID8 | 1.80E-233 | 774.1 | 2.10E-233 | copper resistance system multicopper oxidase [*Dyella* sp. AD56] |
| DYST_01779 | lcced.SFID9 | 1.20E-163 | 543.8 | 1.50E-163 | multicopper oxidase domain-containing protein [*Dyella* sp. AD56] |
| DYST_03157 | lcced.SFID9 | 2.20E-138 | 460.4 | 2.90E-138 | multicopper oxidase domain-containing protein [*Dyella* sp. ASV24] |
| DYST_03158 | lcced.SFID9 | 2.80E-18 | 64.2 | 2.80E-18 | multicopper oxidase domain-containing protein [*Dyella* sp. AD56] |
| ***D. terrae* KACC 12748** | | | | | |
| lcl\|SIZZ01000001.1_prot_TBR39859.1_1299 | lcced.SFID8 | 4.00E-238 | 789 | 4.70E-238 | [locus_tag=EYV96_06650] [protein=copper resistance system multicopper oxidase] [protein_id=TBR39859.1] [location=complement(1487352..1489052)] [gbkey=CDS] |
| lcl\|SIZZ01000001.1_prot_TBR40429.1_1928 | lcced.SFID15 | 1.00E-44 | 151 | 1.40E-44 | [gene=nirK] [locus_tag=EYV96_09810] [protein=nitrite reductase, copper-containing] [protein_id=TBR40429.1] [location=2206374..2207891] [gbkey=CDS] |

**Supplementary Table 9**. HMMER annotation of lignin-degrading enzymes containing multicopper oxidase domains in the *D. terrae* strain Ely Copper Mine and *D. terrae* KACC 12748 genomes using the LccED superfamilies (sfid) followed by CD Search. Genes annotated by sfid, E-values, sequence scores, copper oxidase domain e-values, and gene descriptions are shown.

| gene name | query_name | sequence_evalue | sequence_score | best_domain_evalue | description |
| --- | --- | --- | --- | --- | --- |
| lcl\|NFZS01000001.1_prot_RAO76932.1_625 | lcced.SFID15 | 4.50E-44 | 149 | 6.40E-44 | [locus_tag=CA260_03195] [protein=nitrite reductase, copper-containing] [protein_id=RAO76932.1] [location=733968..735485] [gbkey=CDS] |
| lcl\|NFZS01000001.1_prot_RAO77601.1_1373 | lcced.SFID8 | 2.00E-234 | 776.9 | 2.40E-234 | [locus_tag=CA260_06965] [protein=copper resistance protein CopA] [protein_id=RAO77601.1] [location=1565349..1567076] [gbkey=CDS] |
| lcl\|NFZS01000004.1_prot_RAO75164.1_2706 | lcced.SFID12 | 1.80E-101 | 338.3 | 2.00E-101 | [locus_tag=CA260_13765] [protein=copper oxidase] [protein_id=RAO75164.1] [location=complement(255058..256518)] [gbkey=CDS] |
| lcl\|NFZS01000004.1_prot_RAO75952.1_3008 | lcced.SFID9 | 6.20E-163 | 541.1 | 7.70E-163 | [locus_tag=CA260_15295] [protein=bilirubin oxidase] [protein_id=RAO75952.1] [location=complement(588637..590211)] [gbkey=CDS] |
| lcl\|NFZS01000007.1_prot_RAO74448.1_4031 | lcced.SFID9 | 5.30E-168 | 557.8 | 6.60E-168 | [locus_tag=CA260_20450] [protein=bilirubin oxidase] [protein_id=RAO74448.1] [location=complement(71670..73316)] [gbkey=CDS] |

**Supplementary Table 10**. HMMER annotation of lignin-degrading enzymes containing multicopper oxidase domains in the *D. jiangningensis* FCAV SCS01 genome using the LccED followed by CD Search. Genes annotated by sfid, E-values, sequence scores, copper oxidase domain e-values, and gene descriptions are shown.

| Class of natural product | From nucleotide | To nucleotide | Core biosynthetic genes | Most similar known cluster | % similarity |
| --- | --- | --- | --- | --- | --- |
| *D. terrae Ely Copper Mine* | | | | | |
| *Terpene* | *602,334* | *623,508* | *1* | *Malleobactin A–D* | *11%* |
| *Lanthipeptide class II- type 1 polyketide-nonribosomal peptide* | *1,066,396* | *1,127,084* | *3* | *Capsular polysaccharide* | *3%* |
| *Terpene* | *1,950,940* | *1,971,779* | *5* |  |  |
| *RiPP* | *3,072,432* | *3,082,445* | *1* |  |  |
| *Arylpolyene* | *3,611,586* | *3,652,785* | *9* | *Xanthomonadin I* | *28%* |
| *RiPP* | *3,795,469* | *3,806,323* | *1* |  |  |
| *D. terrae KACC 12748* | | | | | |
| *Thiopeptide* | *198,423* | *22,070* | *2* |  |  |
| *Arylpolyene* | *1,393,453* | *1,442,039* | *3* |  |  |
| *Terpene* | *1,551,631* | *1,572,485* | *1* |  |  |
| *Arylpolyene* | *115,984* | *157,180* | *1* | *Xanthomonadin I* | *35%* |
| *type 1 polyketide-nonribosomal peptide* | *195,428* | *247,342* | *2* | *Capsular polysaccharide* | *3%* |

**Supplementary Table 11**. Six biosynthetic gene clusters (139 genes) identified by antiSMASH version 6.0.1. RiPP = ribosomally synthesized and posttranslationally modified peptide product. A total of 20 annotated genes out of 4,841 genes.

**Supplementary Table 12**. See supplementary material file (Table 4.XLSX) for BacMet annotations of *Dyella* genomes.

| Contigs | 1 |  |  |  |  |  |  |  |  |  |  |  |  |  |  |  |  |  |  |  |
| --- | --- | --- | --- | --- | --- | --- | --- | --- | --- | --- | --- | --- | --- | --- | --- | --- | --- | --- | --- | --- |
| Total Genes | 4841 |  |  |  |  |  |  |  |  |  |  |  |  |  |  |  |  |  |  |  |
| Core Essential Genes | 490 |  |  |  |  |  |  |  |  |  |  |  |  |  |  |  |  |  |  |  |
| Total BGC Hits | 6 |  |  |  |  |  |  |  |  |  |  |  |  |  |  |  |  |  |  |  |
| Known Resistance Models | 37 |  |  |  |  |  |  |  |  |  |  |  |  |  |  |  |  |  |  |  |
| Gene Duplication | 31 |  |  |  |  |  |  |  |  |  |  |  |  |  |  |  |  |  |  |  |
| BGC Proximity | 20 |  |  |  |  |  |  |  |  |  |  |  |  |  |  |  |  |  |  |  |
| Phylogeny/ HGT | 54 |  |  |  |  |  |  |  |  |  |  |  |  |  |  |  |  |  |  |  |
| 2 or more | 15 |  |  |  |  |  |  |  |  |  |  |  |  |  |  |  |  |  |  |  |
| 3 or more | 1 |  |  |  |  |  |  |  |  |  |  |  |  |  |  |  |  |  |  |  |
|  |  |  |  |  |  |  |  |  |  |  |  |  |  |  |  |  |  |  |  |  |

**Supplementary Table 13.** ARTs (https://arts3.ziemertlab.com) annotated contigs using Gamma proteobacteria reference set.

**References**

Edgar, R.C. (2004). MUSCLE: multiple sequence alignment with high accuracy and high throughput. *Nuc Acids Res* 32**,** 1792-1797.

Kalyaanamoorthy, S., Minh, B.Q., Wong, T.K.F., Von Haeseler, A., and Jermiin, L.S. (2017). ModelFinder: fast model selection for accurate phylogenetic estimates. *Nat Methods* 14**,** 587-589.

Minh, B.Q., Schmidt, H.A., Chernomor, O., Schrempf, D., Woodhams, M.D., Von Haeseler, A., and Lanfear, R. (2020). IQ-TREE 2: new models and efficient methods for phylogenetic inference in the genomic era. *Mol Biol and Evol* 37**,** 1530-1534.
